# Obesity impact on leukocyte telomere shortening and immune aging assessed by Mendelian randomization and transcriptomics analysis

**DOI:** 10.1038/s41598-025-16817-5

**Published:** 2025-08-23

**Authors:** Zhijun Feng, Jiacheng Li, Huimin Zhang, Shupeng Liu, Yinghui Wang, Meijuan Zhou, Zhenhua Ding, Lin Xiao

**Affiliations:** 1 Postdoctoral Innovation Practice Base, Jiangmen Central Hospital, Southern Medical University, Jiangmen, 529030 Guangdong China; 2 Department of Radiation Medicine, Guangdong Provincial Key Laboratory of Tropical Disease Research, School of Public Health, Southern Medical University, Guangzhou, 510515 China; 3 Department of Radiation Oncology, Jiangmen Central Hospital, Jiangmen, 529030 Guangdong China

**Keywords:** Immune cell senescence, Leukocyte telomere length, Obesity index, Mendelian randomization, Body mass index, Body fat percentage, Waist circumference, Waist-hip ratio, Data mining, Genetic association study

## Abstract

Obesity and aging are key research topics in contemporary biomedical science. While studies have explored the effects of obesity on various health indicators, the precise mechanisms through which obesity may affect leukocyte telomere length (LTL)-and whether this impact contributes to accelerated immune cell senescence-remain unclear and warrant further investigation. In this study, we employed single nucleotide polymorphisms (SNPs) associated with four obesity indices—body mass index (BMI), body fat percentage (BFP), waist circumference (WC), and waist-hip ratio (WHR)—as instrumental variables (IVs) to assess the causal relationship between these indices and LTL through Mendelian randomization (MR) analysis. Additionally, we analyzed transcriptome sequencing data from peripheral blood mononuclear cells (PBMCs) across three groups: lean individuals, individuals with obesity before undergoing bariatric surgery, and individuals with obesity after surgery, and focus on the expression changes of cellular senescence and telomere dynamics related genes in PBMCs of individuals with obesity before and after weight loss intervention. The results showed a negative causal relationship between BMI (*B*=-0.04, *P* < 0.0001), BFP (*B*=-0.06, *P* < 0.0001) and LTL without being impacted by lipid profiles and T2D. The negative causal relationship between WC (*B*=-0.04, *P* < 0.0001) and LTL may be dependent on lipid levels, but not on T2D. WHR had no significant causal relationship (*P* > 0.05). Transcriptomic analysis further revealed that individuals with obesity had higher expression of cellular senescence-related genes such as *ID2*, *LMNA*, and *TENT4B* in PBMCs compared to lean individuals, with expression levels of these genes significantly decreasing after bariatric surgery. These findings underscore the detrimental impact of obesity on telomere attrition and immune cell senescence, highlighting the potential benefits of obesity management for slowing the biological process of cellular and immune aging.

## Introduction

Given the global rise in obesity prevalence, weight management has become a key public health priority^1–3^. Body mass index (BMI) is widely used as a primary indicator to assess weight status^4^. It is commonly classified into four categories: overweight (25.0–29.9 kg/m²), obesity class Ⅰ (30.0–34.9 kg/m^2^), class Ⅱ (35.0–39.9 kg/m^2^), and class Ⅲ (≥ 40.0 kg/m^2^)^5^. Despite its widespread use in clinical and public health settings, BMI does not account for differences in body composition, and thus may not provide a comprehensive assessment of individual health risks^6,7^. Body fat percentage (BFP), an indicator that directly measures the content of body fat, offers a more comprehensive view of body composition^8^. Abdominal adiposity assessment represents a crucial component of anthropometric evaluation, as visceral fat accumulation is recognized as more metabolically active and pathogenic compared to subcutaneous adiposity^9,10^. Two complementary indicators are commonly employed for this assessment: waist-hip ratio (WHR) and waist circumference (WC). WHR evaluates the distribution pattern of abdominal fat by comparing waist and hip measurements, providing insights into central versus peripheral fat distribution^11^. WC, measured independently of height, serves as a direct indicator of abdominal fat accumulation and visceral adiposity^12^. Both measurements are strongly associated with increased risks of cardiovascular diseases, type II diabetes, and metabolic syndrome, with larger values indicating greater pathogenic potential^13–15^. The clinical significance of these abdominal adiposity markers lies in their ability to identify individuals at elevated risk for obesity-related comorbidities, even when BMI appears normal.

The impact of obesity on biological aging has garnered increasing research attention. Studies indicated that individuals with obesity tend to exhibit more apparent signs of aging compared to those who are not obese at the same age^16,17^. Telomeres are repetitive sequences at chromosome ends that shorten with each cell division, ultimately triggering cellular senescence or apoptosis upon reaching a critical threshold^18,19^. As such, leukocyte telomere length (LTL) is widely recognized as a promising biomarker of biological aging^20,21^. Obesity-related telomere shortening, specially in female smokers, was first highlighted by Valdes et al. in 2005^22^. Additionally, a meta-analysis confirmed a negative association between BMI and LTL in adults^23^. In 2017, a review on the fat mass and obesity-associated (FTO) genes and its related single nucleotide polymorphisms (SNPs) suggested that telomere attrition may be influenced by obesity-related inflammation, oxidative stress, and FTO-associated pathways^24^. Another study confirmed that BMI significantly influences LTL, partly via obesity-induced inflammation and partly through direct, inflammation-independent mechanisms^25^. A meta-analysis further demonstrated that longer LTL is associated with lower WC in adults^26^. In 2024, a Mendelian randomization (MR) analysis provided genetic evidence supporting a causal relationship between overweight status and accelerated aging, including LTL shortening and reduced life expectancy^27^. However, However, this study did not perform detailed BMI stratification or include additional adiposity indicators such as BFP and WC, limiting a more comprehensive assessment of obesity’s impact on aging.

Despite reported associations between obesity and telomere shortening, causal relationships remain unclear due to confounding and limited exposure scope. Given the role of LTL in immune aging, we conducted an MR and transcriptome-based analysis to elucidate the effects of obesity on LTL and immune cell senescence. MR analysis employs genetic variants as instrumental variables (IVs), providing a robust framework to mitigate confounding and assess causal relationships between exposures and health outcomes^28^. This method takes advantage of the random distribution of natural genetic variations, helping to overcome issues of reverse causation and confounding factors that are unresolvable in traditional observational studies^29^. In this study, we selected four types of obesity indices (BMI^30^, BFP, WHR, and WC^31^ as exposures and LTL^32^ as the outcome. Subgroup analysis was performed on the four levels of BMI (overweight, obesity class I-Ⅲ)^5^ and on body fat distribution in various parts (arms, legs, and trunk). Furthermore, we analyzed transcriptomic sequencing data from peripheral blood mononuclear cells (PBMCs) obtained from three groups: lean individuals (mean BMI = 21 kg/m²), individuals with obesity prior to bariatric surgery (mean BMI = 44 kg/m²), and the same individuals three months after surgery (mean BMI = 36 kg/m²). The analysis focused on expression changes in genes related to cellular senescence and telomere dynamics in PBMCs before and after the weight loss intervention. This study aims to elucidate the potential causal associations between obesity indices and LTL, while investigating the impact of obesity on PBMC functionality and senescence. Our findings will provide insights into the interconnections between obesity and both biological aging and immunological aging.

## Materials and methods

### Study design and data source

#### MR analysis of genome-wide association studies (GWAS) data

**Fig. 1:**
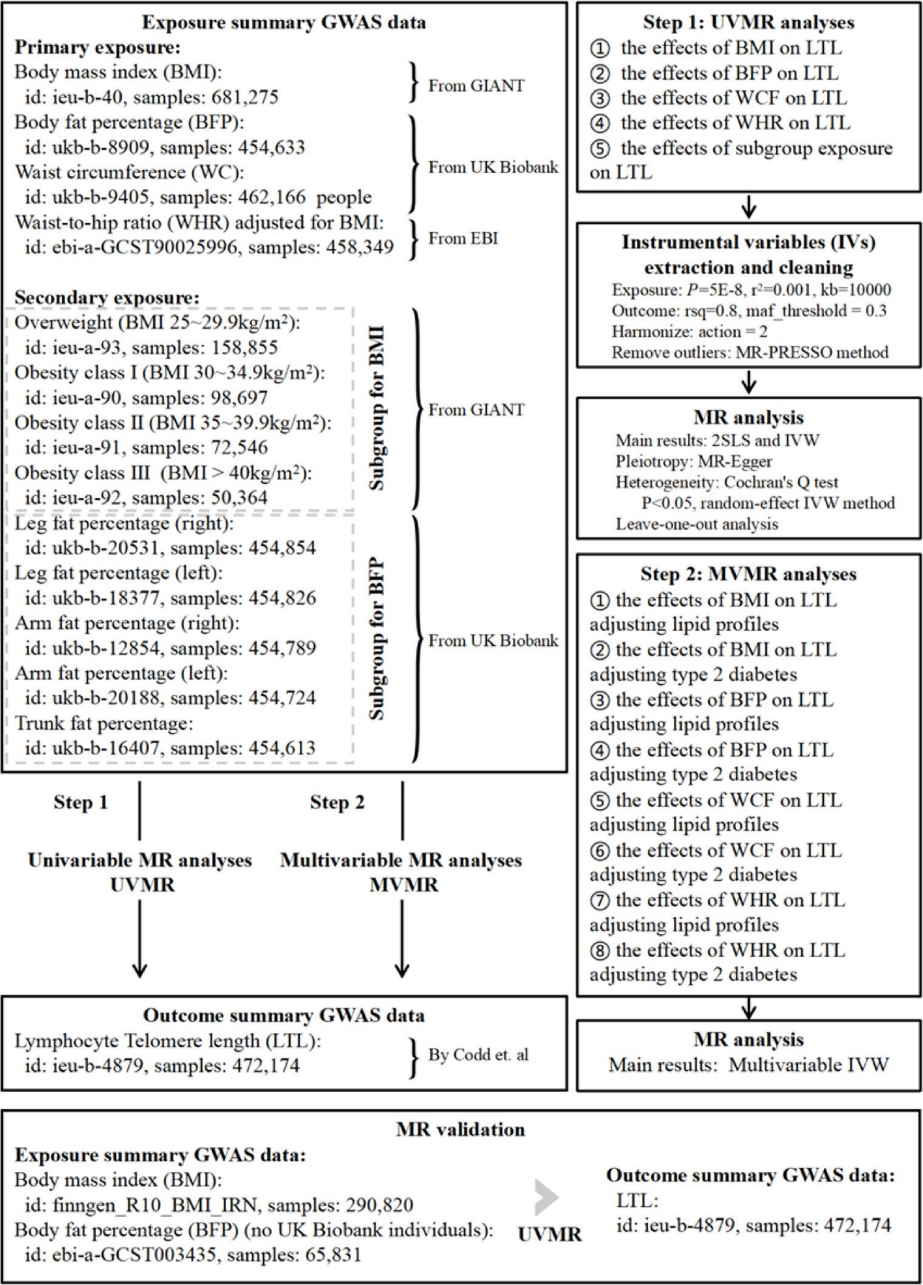
Flow chat for the Mendelian randomization (MR) analysis and the details of data source.

This is a Two-sample MR study following the Strengthening the Reporting of Observational Studies in Epidemiology Using Mendelian Randomization (STROBE-MR Statement) guidelines^33^. We obtained data from the openGWAS database (https://gwas.mrcieu.ac.uk/). Following this, we filtered the data to include only samples with the largest sample sizes and those originating from the same ethnic population for subsequent analysis. We selected four datasets (BMI, BFP, WC, and WHR, Fig. 1) related to obesity indices as the primary exposure variables, and one dataset on LTL as the outcome variable. Additionally, we considered nine datasets (Fig. 1) as secondary exposure variables, which were categorized into four subgroups based on BMI and five subgroups based on BFP. Based on these datasets, we developed a comprehensive framework for MR analysis to investigate the causal relationship between weight and aging. This study employed both univariate MR (UVMR) and multivariate MR (MVMR) analyses. Additionally, we conducted a validation MR analysis, utilizing BMI data from the FinnGen database^34^ and BFP data from samples external to the UK Biobank^35^ (Fig. 1). For the MR analysis to be robust, three key assumptions regarding IVs are essential^36^: First, IVs must demonstrate a robust correlation with the exposure variable. Second, IVs should remain unassociated with any confounding factors that simultaneously affect both the exposure and the outcome. Third, the effects of IVs on the outcome should be exclusively mediated through the exposure, without involving any indirect pathways. Given that the data for this study were sourced from a publicly accessible statistical dataset, no additional ethical approval or informed consent was necessary. Figure 1 provides a comprehensive description of the dataset and the MR analysis procedure, including details on GWAS identification, sample size, traits, and the criteria for IVs selection.

### Transcriptome sequencing analysis of PBMCs

We obtained transcriptomic data from the Gene Expression Omnibus (GEO) database (GSE32575^37^, which includes PBMC mRNA sequencing data from 6 lean individuals and 18 individuals with obesity, each sampled both before and 3 months after bariatric surgery. First, we identified differentially expressed genes (DEGs) among the groups through differential expression analysis and assessed the potential biological processes and signaling pathways associated with these genes. Given that all samples were derived from peripheral blood with relatively consistent cell types, and that the comparisons involved pre- and post-surgery samples from the same individuals, we adopted a relatively inclusive threshold to maximize the detection of meaningful transcriptional changes. Specifically, DEGs were defined as those with an absolute log_2_ fold change (FC) > 0.5 and an adjusted *P*-value < 0.05. Then, using the Molecular Signatures Database (MSigDB), we compiled genes related to cellular senescence and telomere dynamics and evaluated the expression trends of these genes in individuals with obesity before and after bariatric surgery, aiming to clarify the impact of obesity on the aging process of PBMCs.

### IVs obtaining and data cleaning

Based on Fig. 1, we identified IVs associated with exposure using the R package ‘TwoSampleMR’^38^. Missing EAFs were supplemented using data from 1000 Genomes Project^39^. Subsequently, the *F* value for each IV was calculated (*F* = *beta*^2^/*se*^2^), and a threshold *F* value of 10 was established for inclusion in the analysis^40,41^. The data cleaning procedure included the following steps: ⑴ Removal of confounding IVs, specially those related to LTL, tobacco use or smoking intake, which were identified using ‘LDtrait’ database (https://ldlink.nci.nih.gov/?tab=ldtrait)^42^. ⑵ Data harmonization. The ‘TwoSampleMR’ R package was employed to identify outcome-related IVs, utilizing parameters such as proxies set to true (proxies = T), a linkage disequilibrium threshold (rsq) of 0.8, and a minor allele frequency (MAF) threshold of 0.3^43,44^. Subsequently, these IVs were then harmonized with the exposure data. ⑶ Outliers removal. An IV with a *P*-value less than 0.05, as determined by the ‘RadialMR’ R package, was classified as an outlier^45^.

### MR analysis

MR analysis encompassed both UVMR and MVMR analyses. The UVMR analysis was performed utilizing the “TwoSampleMR” R package^38^, and it investigated the causal effects of four primary exposures and nine subgroup exposures on LTL. The MR analysis integrated five methodologies^46^: MR-Egger, weighted median (WM), inverse-variance weighting (IVW), simple mode, and weighted mode, in conjunction with the two-stage least squares (2SLS) method, to evaluate the impact of various exposures on LTL^47,48^. Causality determination adhered to the following criteria^29,49^: ① The causal effect estimates (*B* values, also referred to as *β* values) derived from the five MR methods and the 2SLS method must demonstrated directional consistency, with all values either exceeding (indicating a positive effect) or all being less than 0 (indicating an inverse effect). ② Statistical significance was determined based on a Bonferroni-corrected threshold of *P* < 0.003 (0.05/15), accounting for the 15 exposure traits analyzed in the MR framework^50^.

Considering the potential impact of prevalent metabolic conditions — particularly dyslipidemia and type 2 diabetes (T2D) on — telomere biology, we applied MVMR analysis using the R package “MVMR” to adjust for these confounders^51^. The covariates included key lipid profile traits—high-density lipoprotein (HDL) cholesterol, low-density lipoprotein (LDL) cholesterol, triglycerides (TG), and total cholesterol (TC)—as well as type 2 diabetes (T2D). Summary-level GWAS data for all covariates were obtained from the OpenGWAS database, primarily derived from the UK Biobank cohort. The corresponding data identifiers and sample sizes were as follows: TC (ebi-a-GCST90025953, *n* = 437,878), HDL cholesterol (ieu-b-109, *n* = 403,943), LDL cholesterol (ieu-b-110, *n* = 440,546), TG (ieu-b-111, *n* = 441,016), and T2D (ebi-a-GCST006867, *n* = 655,666). Subsequently, we re-estimated the causal effects of obesity-related indices on LTL after adjusting for these covariates. Effect estimates were derived using the inverse-variance weighted (IVW) method, with statistical significance set at *P* < 0.05.

### Sensitivity analysis

Sensitivity analysis entails examining heterogeneity and assessing pleiotropic effects through the application of Cochran’s Q test and MR‒Egger methods, respectively^52,53^. The MR‒Egger intercept test was employed to evaluate horizontal pleiotropy, with a significance threshold set at *P* < 0.05^54,55^. For the IVW method, a fixed-effects model was utilized in the absence of heterogeneity, where as a random-effects model was adopted when heterogeneity was present (*P*_Q test_<0.05)^52^. Funnel plots and scatter plots derived from MR analysis were utilized to visually evaluate the extent of pleiotropy and heterogeneity.

### Cross validation MR analysis

We re-evaluated the influence of BMI and BFP on LTL utilizing composite GWAS data from different populations. The BMI dataset was derived from the FinnGen database^34^, where as a smaller dataset was selected for BFP, which notably did not contain any samples from the UK Biobank^35^. As a result, these two datasets exhibited no significant sample overlap with the outcome data employed in our current analysis. In accordance with the principles of MR analysis, sample duplication could introduce unpredictable bias into the results. Thus, to confirm that BMI and BFP are causally related to LTL, we re-ran a two-sample UVMR analysis using these datasets in accordance with the quality control conditions mentioned in our methodological steps.

### Transcriptomic analysis of PBMCs

In the transcriptomic analysis of PBMCs, we employed the “limma” package for differential expression analysis^56^. We focused on comparing the gene expression profiles between individuals with obesity before bariatric surgery and lean individuals, as well as the changes in PBMCs gene expression in individuals with obesity before and after bariatric surgery. Subsequently, we used the “clusterProfiler” package for gene enrichment analysis of the DEGs^57^, including gene ontology (GO)^58^ and kyoto encyclopedia of genes and genomes (KEGG) pathway analyses^59^, and conducted gene set enrichment analysis (GSEA) based on the overall differential expression results^57^. Additionally, we retrieved and extracted genes related to cellular senescence and telomere dynamics from the the molecular signatures database (MSigDB) to evaluate the expression trends of these genes in individuals with obesity before and after bariatric surgery^60^. In this analysis, since all samples were derived from PBMCs, and both lean and individuals with obesity represent relatively common population proportions, a revised criterion was applied to better assess the effects of obesity on PBMCs. Differential gene selection was conducted using a threshold of |logFC|>0.5 and P-value < 0.05.

## Results

### The causal relationship between BMI, BFP, WC, WHR and LTL

**Fig. 2:**
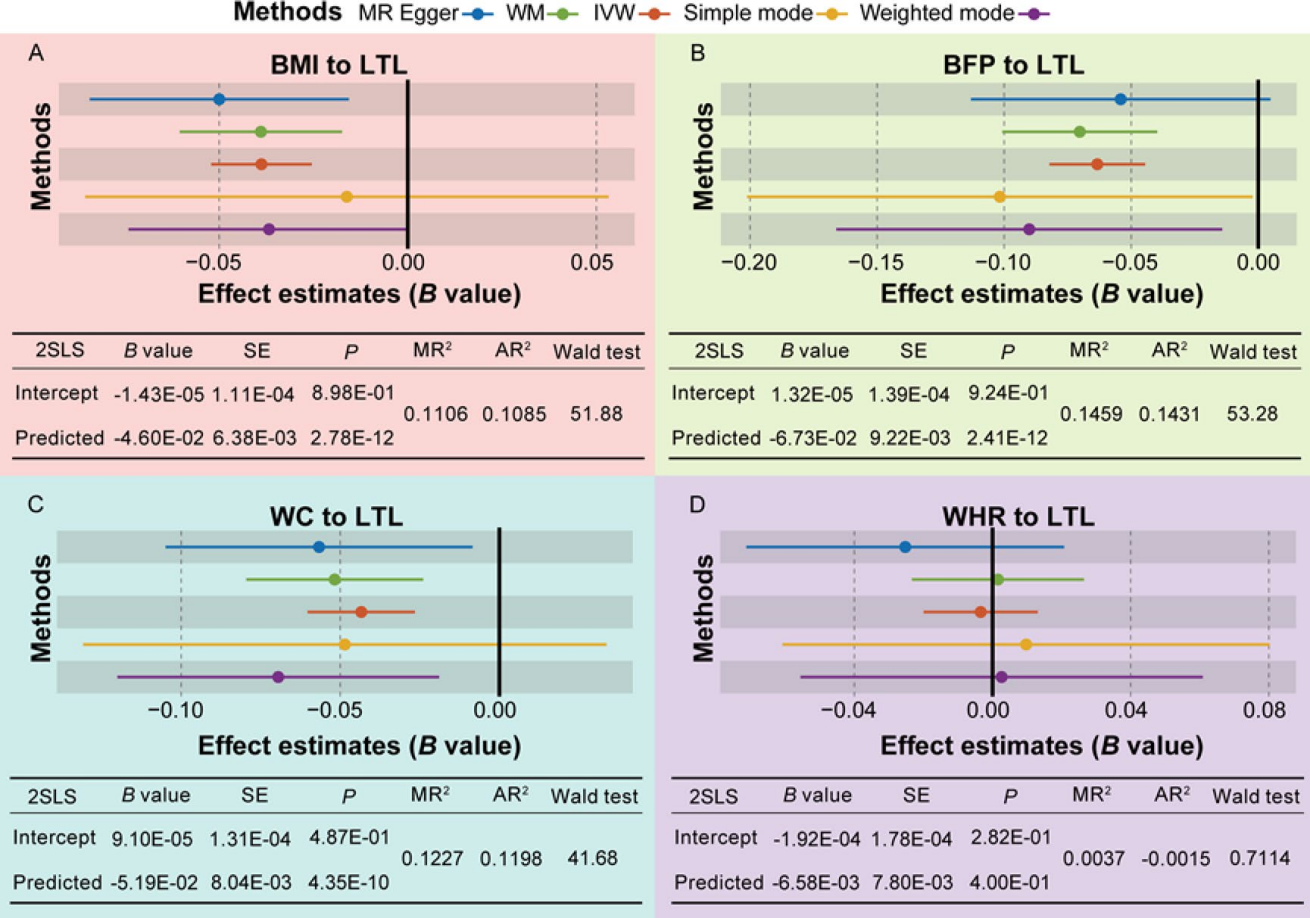
Results of Mendelian randomization and two-stage least squares (2SLS) methods on the causal effects of body mass index (BMI), body fat percentage (BFP), waist circumference (WC), and waist-hip ratio (WHR) on lymphocyte telomere length (LTL). WM, weighted median; IVW, inverse-variance weighting; SE, standard error; MR^2^, multiple R-squared; AR^2^, adjusted R-squared.

SNPs associated with the four primary exposures were extracted as IVs. Detailed information regarding these IVs can be found in Table S1 to Table S4 (Table S1 for BMI, Table S2 for BFP, Table S3 for WC, and Table S4 for WHR). the average F values for BMI, BFP, WC, and WHR were 72.84, 58.26, 57.28, and 79.66 respectively. MR analysis results indicate a significant causal relationship is evident between BMI (*B*= – 0.04, *P*_IVW_=1.14E-08, Fig. 2A), BFP (*B*=-0.06, *P*_IVW_=4.48E-11, Fig. 2B), WC (*B*=-0.04, *P*_IVW_=4.84E-07, Fig. 2C), and LTL. However, no significant causal effect was observed between WHR (*B*=-0.003, *P*_IVW_=0.69, Fig. 2D) and LTL. The detailed results of the MR analysis are presented in Table S5. A comprehensive examination of the 2SLS results in each direction reveals that the causal effects estimates derived from 2SLS are consistent with MR results. The analysis indicates a statistically significant negative association between LTL and BMI, BFP, and WC. Specifically, for each unit increase in BMI, there is a 0.046 unit decrease in LTL (*B*=-0.046, *P* = 2.78E-12, Fig. 2A), which accounts for approximately 11% of the variability in LTL (multiple R-squared (MR^2^) = 0.1106, adjusted R-squared (AR^2^) = 0.1085, Fig. 2A). Similarly, each unit increase in BFP corresponds to a 0.067 unit decrease in LTL (*B*=-0.067, *P* = 2.41E-12, Fig. 2B), explaining approximately 14% of the variance (MR^2^ = 0.1459, AR^2^ = 0.1431, Fig. 2B). Additionally, each unit increase in WC is associated with a 0.052 unit decrease in LTL (*B*=-0.052, *P* = 4.35E-10, Fig. 2C), accounting for approximately 12% of the variance (MR^2^ = 0.1227, AR^2^ = 0.1198, Fig. 2C). In contrast, WHR showed no significant causal association with LTL (*P* = 0.40, Fig. 2D). According to this analysis, BMI, BFP, and WC exhibit significant negative correlations with LTL, with statistically significant evidence of reverse causality. Consequently, in studies examining LTL variation, greater emphasis should be placed on BMI, BFP, and WC, as they exert a more substantial influence on LTL.

### Subgroup analysis of the causal effect of BMI on LTL

The SNPs associated with the four BMI subgroups (overweight, obesity class Ⅰ, obesity class Ⅱ, and obesity class Ⅲ) were extracted following established protocols, with detailed information provided in Table S6 to Table S9. For these subgroups, the mean F values for their IVs were 41.03 (overweight), 39.81 (obesity class Ⅰ), 35.85 (obesity class Ⅱ), and 33.39 (obesity class Ⅲ). According to the results of the 2SLS analysis (Table 1), only the obesity class Ⅰ (*P* = 0.014) and class Ⅲ (*P* = 0.015) subgroups exhibited statistically significant results. However, the MR analysis indicated statistically significant results exclusively for obesity class Ⅲ (*P*_IVW_=0.0002, Table S5) in relation to LTL. Although the causal effects on LTL vary across different BMI levels, an increase in BMI is associated with a more pronounced impact on LTL, particularly within the obesity class Ⅲ group. In this subgroup, the 2SLS method reveals that over 50% of the variability in LTL can be attributed to BMI (MR^2^ = 0.5944, AR^2^ = 0.5365, Table 1). Furthermore, the results from MR analysis in this direction are statistically significant, suggesting a potential direct causal relationship between obesity class Ⅲ and shortened LTL. Conversely, for individuals with a BMI below the threshold for obesity class Ⅱ, the relationship between BMI andL LTL shortening does not exhibit statistical significance. LTL shortening is widely recognized as a biomarker of biological aging. This observation underscores the intricate and heterogeneous relationship between LTL and varying degrees of obesity, thereby highlighting the necessity for personalized strategies in addressing obesity-related health concern. Notably, individuals with severe obesity (BMI > 40 kg/m^2^) demonstrate more significant LTL shortening, suggesting that those with higher levels of obesity may undergo more accelerated biological aging and face heightened health risks. Consequently, it is imperative to implement weight management strategies for this population, with the objective of decelerating the rate of LTL shortening and mitigating the potential long-term health risk associated with this process. In addition to enhancing the quality of life for individuals, such interventions could alleviate the burden of obesity-related chronic diseases on public health systems, thereby reducing healthcare costs.

**Table 1:** The two-stage least squares (2SLS) method evaluated the impact of varying levels of body mass index (BMI) on lymphocyte telomere length (LTL). SE, standard error; MR^2^, multiple R-squared; AR^2^, adjusted R-squared. ^a^, *P*_IVW_=0.06; ^b^, *P*_IVW_=0.0002.

Subgroup	Estimate	SE	*P*	MR^2^	AR^2^	Wald test
Overweight (BMI 25 ~ 29.9Kg/m^2^)						
Intercept	5.35E-04	5.75E-04	3.62E-01	0.0271	-0.0135	0.67
Predicted	-6.77E-03	8.28E-03	4.22E-01			
Obesity class I (BMI 30 ~ 34.9Kg/m^2^)						
Intercept	9.02E-05	4.81E-04	8.52E-01	0.1575	0.1341	6.73
Predicted	-1.20E-02	4.64E-03	1.36E-02^a^			
Obesity class Ⅱ (BMI 35 ~ 39.9Kg/m^2^)						
Intercept	4.70E-04	5.11E-04	3.66E-01	0.0934	0.0621	2.99
Predicted	-6.26E-03	3.62E-03	9.46E-02			
Obesity class Ⅲ (BMI > 40Kg/m^2^)						
Intercept	1.98E-05	8.52E-04	9.82E-01	0.5944	0.5365	10.26
Predicted	-1.14E-02	3.56E-03	1.50E-02^b^			

### Subgroup analysis of the causal effect of BFP on LTL

**Fig. 3:**
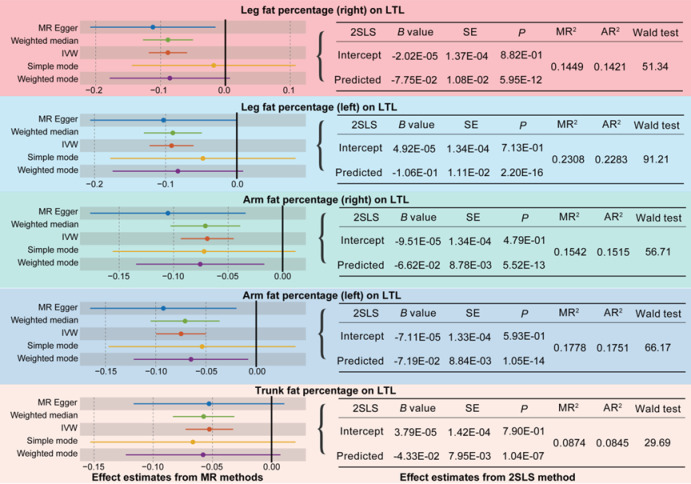
Results of Mendelian randomization (MR) and two-stage least squares (2SLS) methods on the causal effects of subgroup of body fat percentage (BFP) on lymphocyte telomere length (LTL). WM, weighted median; IVW, inverse-variance weighting; SE, standard error; MR^2^, multiple R-squared; AR^2^, adjusted R-squared.

SNPs associated with five BFP subgroups were identified using established criteria, with comprehensive details provided in Table S10 to Table S14. The average F values for the IVs were 54.5 (right leg), 54.81 (left leg), 58.48 (right arm), 59.18 (left arm), and 57.62 (trunk). MR analysis demonstrated an inverse causal relationship between fat percentage and LTL across all five subgroup analyses (Fig. 3 and Table S5, right leg: *B*= – 0.08, *P*_IVW_=4.99E-11; left leg: *B*=-0.097, *P*_IVW_=2.02E-15; right arm: *B*= – 0.058, *P*_IVW_=2.15E-09; left arm: *B*=-0.067, *P*_IVW_=5.56E-12; trunk: *B*=-0.047, *P*_IVW_=6.95E-09). Additionally, the 2SLS analysis corroborated these findings, with causal effect estimates aligning with those from the MR analysis (Fig. 3). Specifically, fat percentage in various body regions accounted for 8–23% of the variability in LTL. Notably, the fat percentage in the trunk exhibited the lowest explanatory power at 8% (MR^2^ = 0.0874, AR^2^ = 0.0845), where as the left leg fat percentage demonstrated the highest explanatory power at 23% (MR^2^ = 0.2308, AR^2^ = 0.2283). Additionally, the results obtained from the 2SLS analysis were statistically significant (Fig. 3, *P* < 0.003). These findings suggest that LTL is uniformly influenced by fat distribution across different regions of the body, underscoring the importance of considering specific fat deposits in studies of cellular aging. The differential explanatory power observed across various anatomical regions indicates a complex interplay between regional fat distribution and telomere dynamics, offering significant implications for personalized interventions targeting obesity-related health issues. This observation highlights the necessity for more detailed research in this domain, which could potentially lead to more effective strategies for mitigating the detrimental effects of adipose tissue accumulation on the aging process.

### MVMR analysis to adjust lipid profiles and diabetes

Given the metabolic interrelationships among adiposity, dyslipidemia, and T2D, and their potential influence on telomere dynamics, we conducted a MVMR analysis to adjust for lipid profiles and T2D when evaluating the causal effects of four primary obesity-related exposures on LTL. The results indicate that BMI remains statistically significant even after adjusting for lipid profiles (Table 2, B=-0.049, *P* = 0.011) and T2D (Table 2, B=-0.028, *P* = 0.017). Similarly, BFP also demonstrates a causal effect after adjustments for lipid profiles (Table 2, B=-0.079, *P* = 0.001) and T2D (Table 2, B=-0.068, *P* = 8.13E-05). Nevertheless, the statistical significance of the relationship between WC and changes in LTL was not observed after adjusting for lipid profiles (Table 2, B=-0.036, *P* = 0.15), but remained significant (Table 2, B=-0.033, *P* = 0.041) after adjusting for T2D alone. Specially, after adjusting for lipid profiles (Table 2, B=-0.011, *P* = 0.51) and T2D (Table 2, B=-0.002, *P* = 0.9), the causal effect of WHR on LTL was not statistically significant. The results of this study suggested that, even after adjusting for lipid profiles and T2D, BMI and BFP continue to have a significant inverse association with LTL, where as the effects of WC and WHR are less conclusive. These findings underscore the significance of BMI and BFP in the aging process of the human body. Given the LTL function as a biological marker of aging, the adverse effects of elevated BMI and BFP on LTL suggest that higher levels of these measures may accelerate the biological aging process. Consequently, maintaining a healthy weight and BFP is essential for decelerating the aging process.

**Table 2:** Multivariate Mendelian randomization (MVMR) analysis to adjust metabolic factors or diseases.

GWAS ID.exposure	Traits.exposure	*N*_snp_	B	SE	*P*
BMI adjusting lipid profiles					
ieu-b-40	BMI	135	– 4.86E-02	1.92E-02	1.14E-02*
ebi-a-GCST90025953	TC levels	40	2.14E-01	1.84E-01	2.44E-01
ieu-b-109	HDL cholesterol	55	– 8.12E-02	6.47E-02	2.09E-01
ieu-b-110	LDL cholesterol	35	– 1.98E-01	1.72E-01	2.48E-01
ieu-b-111	Triglycerides	34	– 4.40E-02	3.95E-02	2.65E-01
BMI adjusting type 2 diabetes					
ieu-b-40	BMI	387	– 2.76E-02	1.15E-02	1.67E-02*
ebi-a-GCST006867	Type 2 diabetes	57	– 3.34E-03	5.07E-03	5.10E-01
BFP adjusting lipid profiles					
ukb-b-8909	BFP	103	– 7.92E-02	2.43E-02	1.10E-03*
ebi-a-GCST90025953	TC levels	94	– 7.67E-03	1.85E-02	6.79E-01
ieu-b-109	HDL cholesterol	116	7.99E-03	1.38E-02	5.62E-01
ieu-b-110	LDL cholesterol	78	2.09E-02	2.21E-02	3.45E-01
ieu-b-111	Triglycerides	98	1.59E-02	1.46E-02	2.73E-01
BFP adjusting type 2 diabetes					
ukb-b-8909	BFP	261	– 6.79E-02	1.72E-02	8.13E-05*
ebi-a-GCST006867	Type 2 diabetes	70	– 3.40E-03	4.99E-03	4.96E-01
WC adjusting lipid profiles					
ukb-b-9405	WC	73	– 3.56E-02	2.47E-02	1.49E-01
ebi-a-GCST90025953	TC levels	91	– 7.25E-03	1.97E-02	7.12E-01
ieu-b-109	HDL cholesterol	118	4.19E-03	1.47E-02	7.75E-01
ieu-b-110	LDL cholesterol	77	3.03E-02	2.35E-02	1.98E-01
ieu-b-111	Triglycerides	102	1.64E-02	1.54E-02	2.88E-01
WC adjusting type 2 diabetes					
ukb-b-9405	WC	253	– 3.25E-02	1.59E-02	4.12E-02*
ebi-a-GCST006867	Type 2 diabetes	71	4.13E-05	5.29E-03	9.94E-01
WHR adjusting lipid profiles					
ebi-a-GCST90025996	WHR	115	– 1.08E-02	1.66E-02	5.14E-01
ebi-a-GCST90025953	TC levels	92	– 3.38E-02	6.17E-02	5.85E-01
ieu-b-109	HDL cholesterol	119	1.57E-02	2.36E-02	5.05E-01
ieu-b-110	LDL cholesterol	73	5.57E-02	6.03E-02	3.56E-01
ieu-b-111	Triglycerides	111	2.46E-02	1.73E-02	1.56E-01
WHR adjusting type 2 diabetes					
ebi-a-GCST90025996	WHR	154	1.89E-03	1.53E-02	9.02E-01
ebi-a-GCST006867	Type 2 diabetes	46	– 2.71E-03	6.01E-03	6.52E-01

*N*_snp_, number of SNPs; *B*, causal effect estimation; BMI, body mass index; TC, total cholesterol; HDL, high density lipoprotein; LDL, low density lipoprotein; BFP, body fat percentage; WC, waist circumference; WHR, waist-hip ratio.

### Sensitivity analysis

**Fig. 4:**
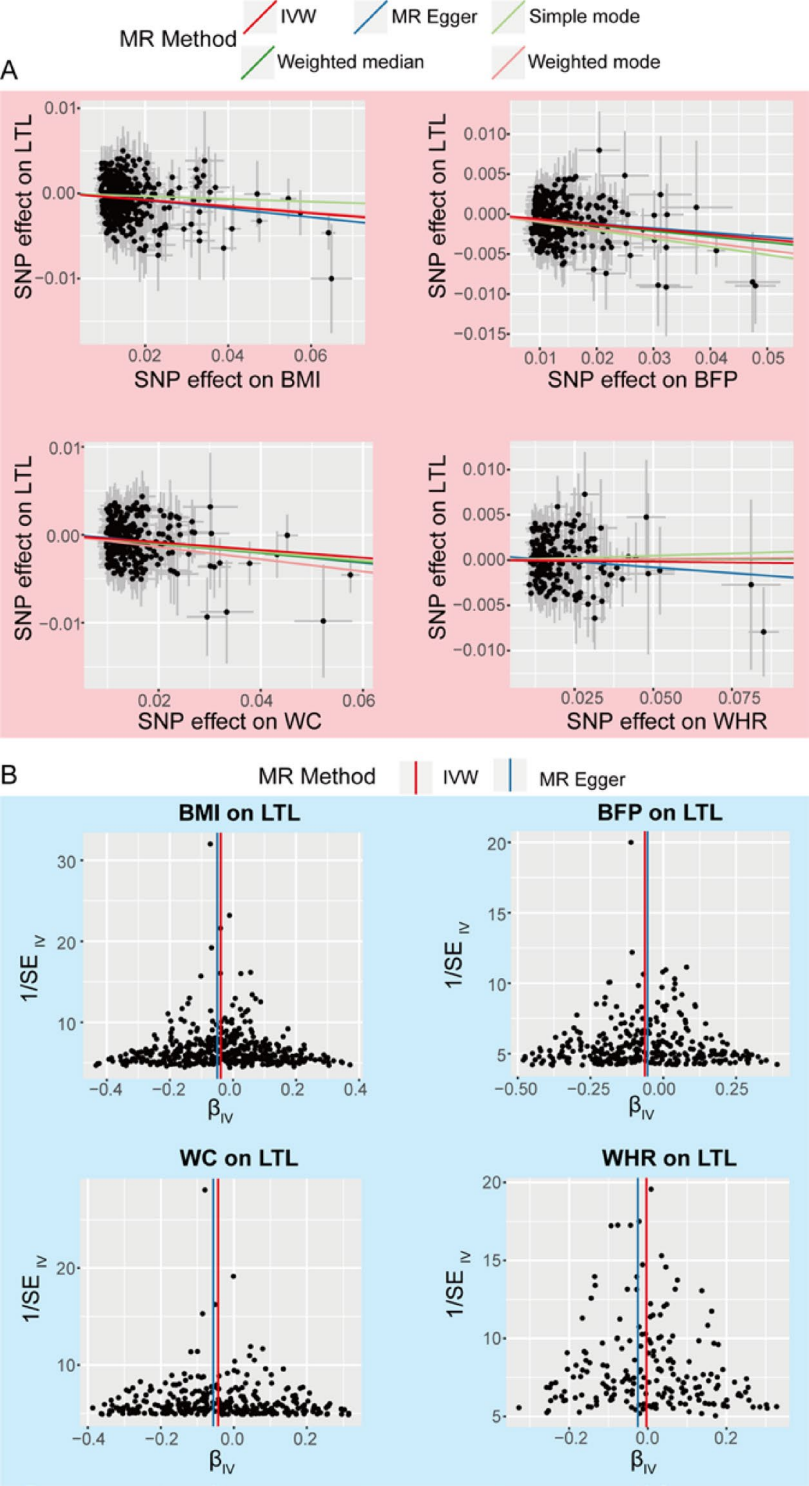
Scatter plots (**A**) and funnel plots (**B**) of causal estimates of TL for 4 primary exposures. BMI, body mass index; BFP, body fat percentage; WC, waist circumference; WHR, waist-hip ratio; LTL, lymphocyte telomere length; IVW, inverse-variance weighting; SE, standard error; SNP, single nucleotide polymorphism.

The sensitivity analysis entailed evaluating heterogeneity using Cochran’s Q test and assessing pleiotropy via the MR-Egger method. The results indicated an absence of statistically significant evidence for either heterogeneity or pleiotropy in the MR analysis of the four primary exposures on LTL, as well as in the analysis of nine additional adiposity-related subgroups, including overweight, obesity classes, and regional BFPs (Table S15). To assess the robustness of our causal estimates, we conducted sensitivity analyses. Leave-one-out analyses were performed to evaluate the stability of causal effect estimates following sequential exclusion of individual SNPs. These analyses revealed no outlying SNPs that substantially influenced the overall causal estimates across all exposure variables (BMI: Figure S1; BFP: Figure S2; WC: Figure S3; WHR: Figure S4), confirming the robustness of our findings. Visual inspection of causal relationships was facilitated through scatter plots (Fig. 4A) and funnel plots (Fig. 4B) for the four primary obesity-related exposures. The scatter plots demonstrate consistent directional effects across instrumental variables, while the funnel plots exhibit symmetrical distribution of effect estimates around the overall causal estimate, indicating absence of small-study effects or bias. Analogous sensitivity analyses were extended to nine additional exposure variables, with corresponding leave-one-out analysis provided in the supplementary materials (Figures S5-S13: overweight [S5], obesity class I [S6], obesity class II [S7], obesity class III [S8], regional fat percentages for right leg [S9], left leg [S10], right arm [S11], left arm [S12], and trunk [S13]). Comprehensive assessment of horizontal pleiotropy was conducted using scatter plots (Figure S14) and funnel plots (Figure S15), which collectively indicate minimal likelihood of pleiotropy-induced bias affecting our causal inferences.

### Validation analysis

**Fig. 5:**
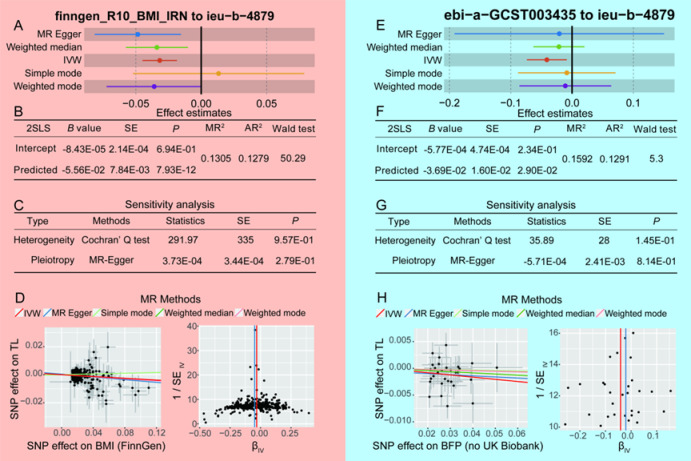
The results of validation Mendelian randomization (MR) analysis. finngen_R10_BMI_IRN, the identification code of body mass index (BMI) from FinnGen database; ieu-b-4879, the identification code of lymphocyte tolemere length (LTL) from openGWAS database; IVW, inverse-variance weighting; 2SLS, two-stage least squares; SE, standard error; MR^2^, multiple R-squared; AR^2^, adjusted R-squared; SNP, single nucleotide polymorphism.

External validation was performed to corroborate the findings of the MR analysis. The data sources were refined, with BMI data derived from the FinnGen database and BFP data obtained from non-UK populations. Combining with the 2SLS method, the MR analysis aimed to establish a causal relationship. Detailed information on the SNPs associated with the two exposures is provided in Table S16 (for BMI) and Table S17 (for BFP). The average *F* values of the IVs were 42.87 for BMI and 28.91 for BFP. The MR analysis (Table S18) revealed inverse causal relationships between both BMI and BFP with LTL (Fig. 5A of MR analysis for BMI on LTL: *B*=-0.032, *P*_IVW_=2.24E-06; Fig. 5B of 2SLS method for BMI on LTL: *B*=-0.056, *P* = 7.93E-12; Fig. 5E of MR analysis for BFP on LTL: *B*=-0.041, *P*_IVW_=1.32E-02; Fig. 5F of 2SLS method for BMI on LTL: *B*=-0.037, *P* = 2.90E-02). The explanatory power of BMI (MR^2^ = 13.05, AR^2^ = 12.79) and BFP (MR^2^ = 15.92, AR^2^ = 12.91) for LTL variation was approximately13% in the validation analysis, demonstrating consistency with the previous findings in this study. Sensitivity analyses in these directions did not yield any statistically significant results (Fig. 5C for BMI, Fig. 5G for BFP). Additionally, neither scatter plots nor funnel plots (Fig. 5D for BMI, Fig. 5H for BFP) revealed any atypical value distributions. The leave-one-out analysis showed that no specific SNP affected the overall effect (Figure S16 for FinnGen database and Figure S17 for ebi-a-GCST003435 data set). These evidences suggest that BMI and BFP negatively impact on LTL, highlighting their significance in the aging process.

### Transcriptomic analysis of PBMCs

**Fig. 6:**
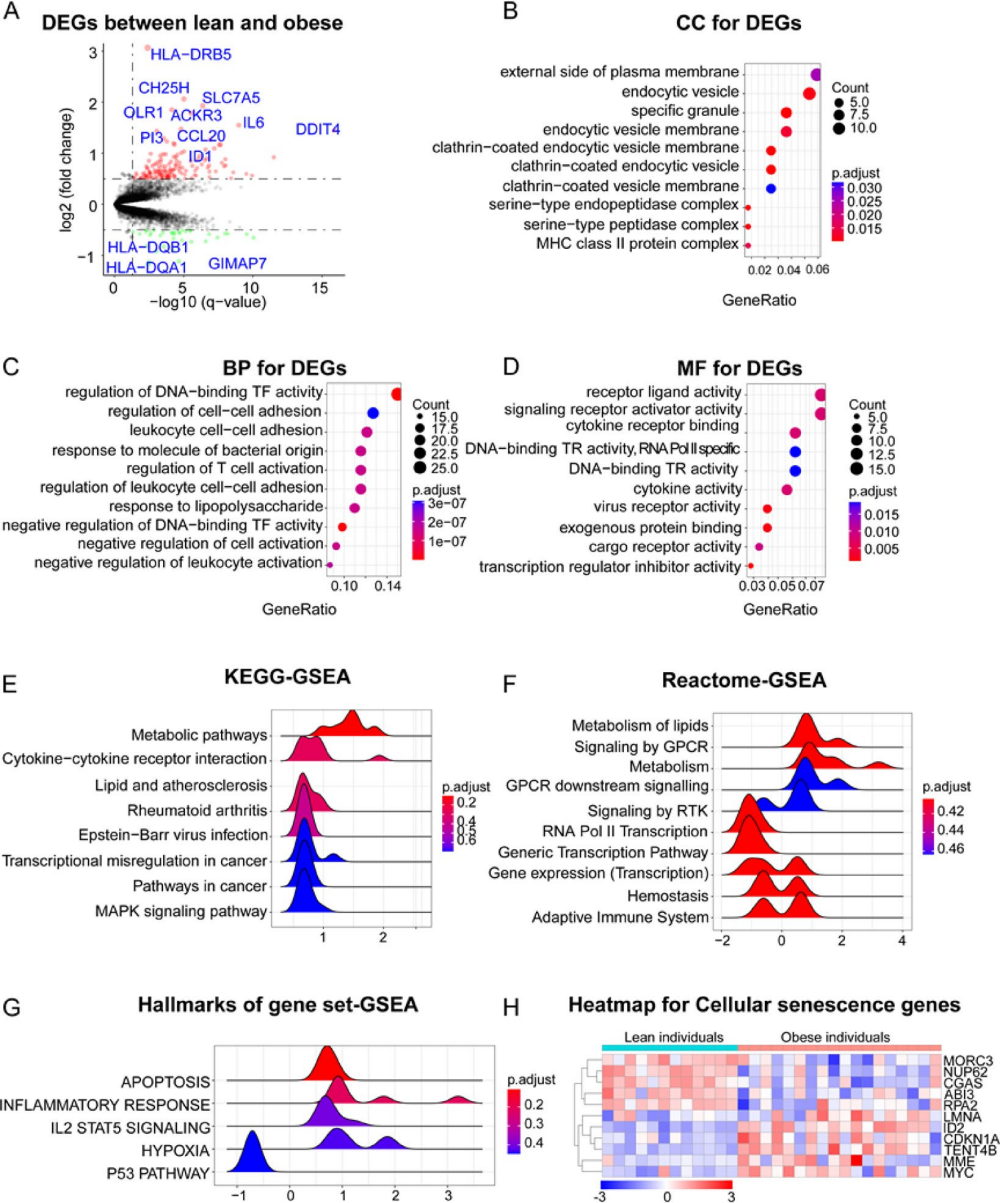
The results of bioinformatics analysis comparing individuals with obesity with lean individuals. (**A**), volcano plot for differentially expressed genes (DEGs) comparing individuals with obesity with lean individuals. Red points represent the genes up-regulated with significance in individuals with obesity. Green points represent the genes down-regulated with significance in individuals with obesity. Gray points represent genes with no significance. (**B**), the results of cellular components(CC) for DEGs. (**C**), the results of biological process(BP) for DEGs. (**D**), the results of molecular function (MF) for DEGs. (**E**), the results of Kyoto Encyclopedia of Genes and Genomes (KEGG) analysis based on gene set enrichment analysis (GSEA). (**F**), the results of Reactome pathway based on GSEA. (**G**), the results of hallmarks of gene set based on GSEA. H, heatmap plot for genes related cellular senescence and telomere dynamics.

Compared to lean individuals, the PBMCs of individuals with obesity exhibited significant gene expression abnormalities (Fig. 6A). According to the predetermined criteria for screening DEGs, a total of 186 DEGs were identified (Table S19). Among these, 150 genes were upregulated, including *HLA-DRB5*, *CH25H*, *SLC7A5*, *OLR1*, *ACKR3*, and *IL6* (Fig. 6A). The remaining 36 genes were downregulated, primarily involving *GIMAP7* and *HLA* family members such as *HLA-DQB1* and *HLA-DQA1* (Fig. 6A). The cellular components (CC) of these DEGs were mainly associated with membrane proteins and vesicles, including locations such as the external side of the plasma membrane, endocytic vesicles, endocytic vesicle membranes, and clathrin-coated endocytic vesicle membranes (Fig. 6B). In terms of biological processes (BP), the DEGs were predominantly involved in the regulation of immune cell activities, including T cell and leukocyte cell-cell adhesion, T cell and leukocyte activation, and DNA-binding transcription factor activity (Fig. 6C). Regarding molecular functions (MF), these genes were mainly associated with receptor-ligand activity, signaling receptor activator activity, cytokine receptor binding, cytokine activity, and transcription regulator inhibitor activity (Fig. 6D). KEGG pathway analysis based on GSEA (Fig. 6E) indicated that, compared to lean individuals, the PBMCs of individuals with obesity were primarily involved in metabolic pathways, cytokine-cytokine receptor interaction, lipid and atherosclerosis, rheumatoid arthritis, and Epstein-Barr virus infection pathways. This suggests that, in individuals with obesity, PBMCs exhibit not only alterations in metabolic status but also activation of inflammatory responses. The Reactome pathway analysis based on GSEA (Fig. 6F) revealed that the PBMCs of individuals with obesity mainly participated in pathways related to lipid metabolism, G Protein-Coupled Receptor (GPCR) signaling, general metabolism, GPCR downstream signaling, Receptor tyrosine kinases (RTK) signaling, RNA polymerase II transcription, generic transcription pathways, gene expression (transcription), hemostasis, and the adaptive immune system. The hallmark gene set analysis using GSEA (Fig. 6G) showed that gene sets such as APOPTOSIS (genes mediating programmed cell death (apoptosis) by activation of caspases), INFLAMMATORY RESPONSE (genes defining inflammatory response), IL2-STAT5 SIGNALING (genes up-regulated by STAT5 in response to IL2 stimulation), and HYPOXIA (genes up-regulated in response to low oxygen levels (hypoxia)) were activated in the PBMCs of individuals with obesity compared to lean individuals, where as the P53 PATHWAY (genes involved in p53 pathways and networks) gene set was suppressed. Finally, we analyzed the expression changes of 352 genes closely related to cellular senescence and telomere dynamics based on the DEGs (Table S20). We found that *MORC3*, *NUP62*, *CGAS*, *ABI3*, and *RPA2* were highly expressed in lean individuals, where as *LMNA*, *ID2*, *CDKN1A*, *TENT4B*, *MME*, and *MYC* were upregulated in the PBMCs of individuals with obesity (Fig. 6H).

**Fig. 7:**
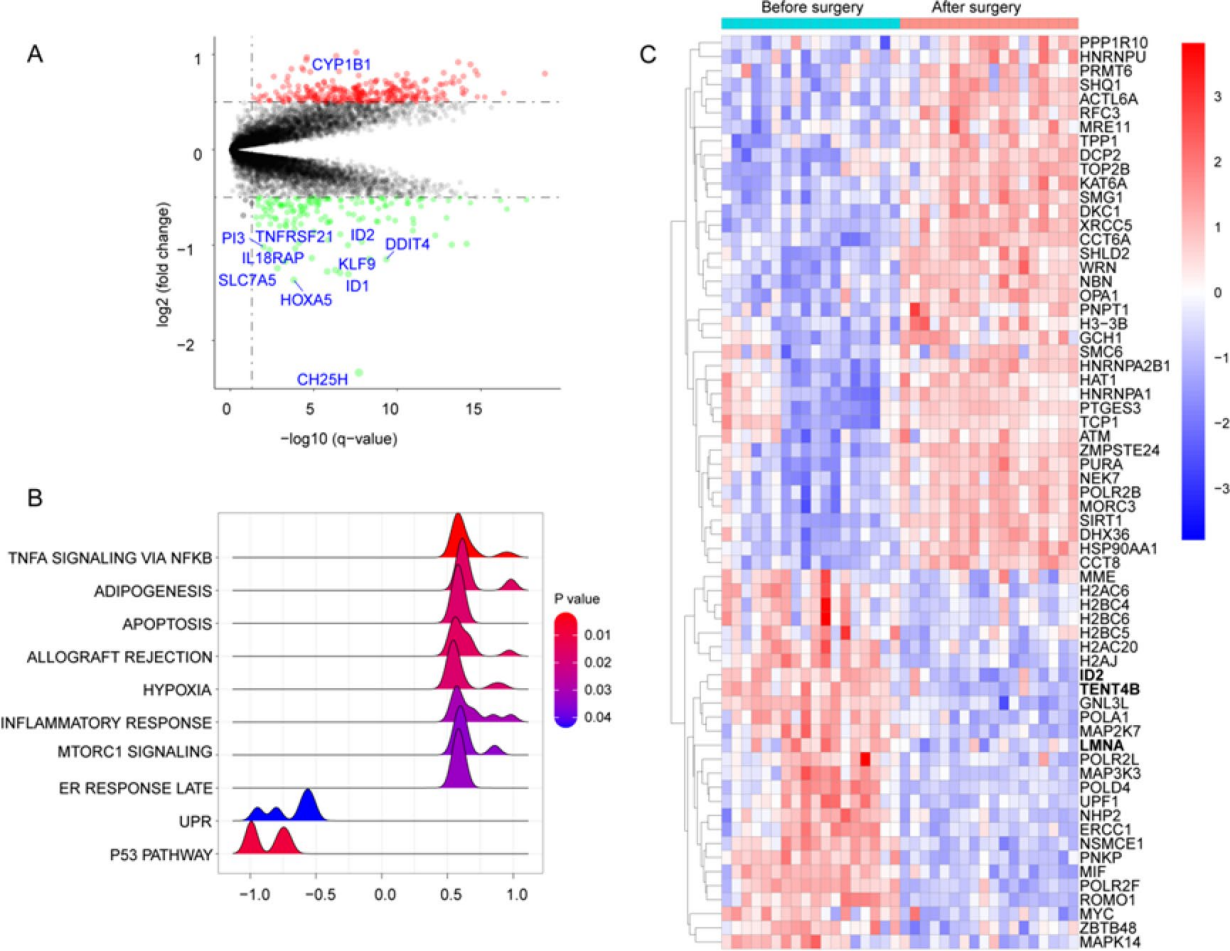
The results of bioinformatics analysis comparing individuals with obesity before bariatric surgery with after bariatric surgery. (**A**), volcano plot for differentially expressed genes (DEGs) comparing individuals with obesity before bariatric surgery with after bariatric surgery. Red points represent the genes up-regulated with significance in individuals with obesity after bariatric surgery. Green points represent the genes down-regulated with significance in obese individuals after bariatric surgery. Gray points represent genes with no significance. (**B**), the results of hallmarks of gene set based on gene set enrichment analysis (GSEA). (**C**), heatmap plot for genes related cellular senescence and telomere dynamics.

Compared to individuals with obesity before bariatric surgery, the PBMCs after surgery exhibited more pronounced changes in gene expression profiles (Fig. 7A). A total of 455 DEGs were identified (Table S21). Among these, 312 genes were upregulated, including *CYP1B1*, *CD164*, *CCR2*, *CAPZA1*, *MAP3K1*, *CHD9*, *HSPA1A*, *EGR2*, and *PTMA* (Fig. 7A, Table S21). The remaining 143 genes were downregulated, primarily including *DDIT4*, *ACKR3*, *SLC7A5*, *IL1R2*, *CA4*, *ZBTB16*, *ID1*, *HOXA5*, and *CH25H* (Fig. 7A, Table S21). The CC of these DEGs was mainly associated with the nuclear envelope, nuclear specks, nuclear membrane, secretory granule lumen, cytoplasmic vesicle lumen, vesicle lumen, ficolin-1-rich granules and their lumens, promyelocytic leukemia (PML) bodies, and euchromatin (Figure S18). In terms of BP, the DEGs were mainly involved in positive regulation of the mitogen-activated protein kinase (MAPK) cascade, regulation of apoptotic signaling pathways, nucleocytoplasmic transport, nuclear transport, and nuclear export (Figure S18). Regarding MF, these genes were primarily associated with DNA-binding transcription factor binding, guanosine triphosphate (GTP) binding, and guanyl nucleotide binding (Figure S18). KEGG enrichment analysis based on “clusterProfiler” indicated that these DEGs participated in pathways such as salmonella infection, lipid and atherosclerosis, and viral protein interaction with cytokines and cytokine receptors (Figure S18). The KEGG pathway analysis based on GSEA (Figure S19) demonstrated that, compared to before surgery, the PBMCs after bariatric surgery showed significant changes in the following signaling pathways: metabolic pathways, viral protein interaction with cytokines and cytokine receptors, cytokine-cytokine receptor interaction, transcriptional misregulation in cancer, lipid and atherosclerosis, pathogenic escherichia coli infection, MAPK signaling pathway, endocytosis, and cancer pathways were activated, where as the herpes simplex virus 1 infection pathway was suppressed. The Reactome pathway analysis based on GSEA (Figure S19) indicated that the PBMCs after surgery mainly involved pathways such as signaling by GPCR, GPCR downstream signaling, neutrophil degranulation, transport of small molecules, and metabolism.

The hallmark gene set analysis using GSEA (Fig. 7B) showed that, compared to before surgery, gene sets such as TNFA SIGNALING VIA NFKB (genes regulated by NF-kB in response to TNF), ADIPOGENESIS (genes up-regulated during adipocyte differentiation), APOPTOSIS, ALLOGRAFT REJECTION (genes up-regulated during transplant rejection), HYPOXIA, INFLAMMATORY RESPONSE, MTORC1 SIGNALING (genes up-regulated through activation of mTORC1 complex), and ESTROGEN (ER) RESPONSE LATE (genes defining late response to estrogen) were significantly activated in the PBMCs after bariatric surgery, where as gene sets such as UNFOLDED PROTEIN RESPONSE (UPR, genes up-regulated during unfolded protein response, a cellular stress response related to the endoplasmic reticulum) and P53 PATHWAY were significantly suppressed. Finally, we evaluated the expression changes of 352 genes closely related to cellular senescence and telomere dynamics based on the DEGs. We confirmed that *LMNA*, *ID2*, and *TENT4B* were significantly upregulated before surgery but showed a marked downregulation trend after surgery (Fig. 7C). Additionally, we found that histone-related genes such as *H2AC6*, *H2BC4*, *H2BC5*, *H2BC6*, and *H2AC20* were also significantly downregulated following bariatric surgery.

## Discussion

In this study, we employed MR coupled with 2SLS analysis to investigate the causal associations between LTL and four obesity indices: BMI, BFP, WC, and WHR. Additionally, we conducted transcriptomic analyses to examine the impact of obesity on immune cell senescence. Through detailed subgroup analyses of BMI and BFP, we identified the most robust predictive indicators. Our comprehensive investigation yielded four key findings that illuminate the relationship between obesity and LTL, as well as its effects on PBMCs.

Firstly, through MR analysis primarily using IVW, combined with the 2SLS method, the results indicated significant negative causal relationships between BMI, BFP, WC, and LTL. And this result was consistently supported by validation analyses across different populations, comprehensively confirming the impact of obesity on the aging process marked by LTL. Additionally, the results of the MVMR analysis indicated that the effect of BMI and BFP on LTL is not significantly influenced by individual lipid levels or the presence of T2D. This not only reveals the mechanism by which obesity may accelerate biological aging at the overall level but also emphasizes the importance of maintaining a healthy weight in delaying the aging process.

Secondly, we observed that different degrees of BMI have varying effects on LTL. Notably, severe obesity (BMI ≥ 40 kg/m^2^) showed the most significant negative impact on LTL. Interestingly, in subgroup analyses of BMI < 40 kg/m^2^, including overweight, obesity class Ⅰ, obesity class Ⅱ, we did not observe significant causal effects on LTL. This finding suggests that the influence of obesity on LTL may have a threshold, only significantly accelerating the biological aging process when BMI exceeds a specific threshold. This result underscores the critical role of preventing and controlling severe obesity in promoting healthy aging, while also providing important references for clinical practice and public health policy formulation.

Thirdly, for the subgroup analysis of BFP revealed an important finding: fat percentages in the upper limbs, lower limbs, and trunk all showed significant negative causal relationships with LTL. This result not only confirms the widespread influence of fat percentage on LTL but also highlights the importance of body fat distribution in assessing obesity-related health risks. Notably, this finding suggests that BFP might be a more comprehensive and sensitive indicator when evaluating the impact of obesity on aging process, as it reflects the consistent influence of fat distribution across different body regions on biological aging. Furthermore, sensitivity analyses for MR analysis did not reveal significant pleiotropy or heterogeneity, further enhancing the reliability and robustness of current research findings.

Fourth, obesity-induced cellular senescence may contribute to telomere-related dysfunction. Since LTL was measured in leukocytes, telomere attrition could potentially impair immune cell function and stability. Through comparative transcriptomic analyses of samples from lean individuals and individuals with obesity (both before and after bariatric surgery), we identified DEGs and BPs associated with obesity and immune senescence. Notably, senescence-associated genes such as *ID2*, *LMNA*, *CDKN1A*, and *CTNNB4* were dysregulated in obesity and partially reversed after surgery. Although these genes are implicated in pathways relevant to cellular aging, their direct roles in telomere maintenance require further validation. Therefore, our findings support a link between obesity and immunosenescence that may involve telomere-associated mechanisms, but further studies are warranted to clarify the mechanistic relationship.

From a clinical perspective, the findings of this study are significant in the following aspects. Firstly, threshold effect of obesity on LTL. Although several studies have confirmed the impact of BMI on LTL^61,62^, most of these studies have focused on overall population-level analyses. Our subgroup analysis, however, demonstrates that this causal effect becomes significant only when BMI exceeds 40 kg/m^2^. This finding challenges the prevailing understanding of BMI’s impact on LTL and suggests the presence of a threshold effect. In other words, mild to moderate obesity may not significantly affect LTL, while severe obesity is the key factor leading to marked telomere shortening. This evidence not only deepens our understanding of the relationship between obesity and biological aging, but also highlights the importance of stratified management of obesity. Secondly, BFP as a critical indicator for assessing obesity-related aging risk. The study found that BFP in the upper limbs, lower limbs, and trunk all showed significant negative causal relationships with LTL, indicating that fat distribution has a broad impact on biological aging. Compared to single measurements like weight or BMI, BFP better reflects the comprehensive distribution of body fat. Therefore, in clinical practice, BFP can serve as a more sensitive indicator for assessing obesity-related health risks and the body’s aging process. Thirdly, our findings have significant clinical implications for obesity management and healthy aging intervention. Transcriptomic analyses demonstrate that obesity-induced immune cell senescence can be reversed through bariatric surgery, as evidenced by the normalization of senescence-associated gene expression markers. These findings not only establish quantifiable benchmarks for clinical intervention but also validate bariatric surgery as a potential therapeutic strategy to mitigate obesity-related immunosenescence, thereby offering evidence-based guidance for personalized obesity management and healthy aging promotion.

Obesity has been increasingly recognized as a significant contributor to accelerated aging, particularly through its association with age-related cellular accumulation^63–65^. While a previous meta-analysis demonstrated a negative correlation between BMI and LTL, it lacked causal evidence due to methodological limitations^23^. Notably, studies across different age groups have shown that both young individuals with obesity and overweight children and adolescents exhibit shorter LTLs^66,67^, with further evidence indicating that maternal pre-pregnancy BMI correlates with shorter LTL in newborns^68^. In recent years, BFP has has emerged as a more precise indicator of obesity, offering direct measurement of adiposity^69^. Analysis of the NHANES database revealed negative correlations between LTL and multiple obesity indices, including BMI, WC, and BFP^70^. Interestingly, gender-stratified analyses have uncovered sex-specific differences, with LTL shortening associated with BFP in boys but not in girls^71^. Collectively, these findings underscore the profound relationship between weight abnormalities and LTL across the human lifespan, from newborns to adults, suggesting obesity’s role in accelerating cellular aging. The broad implications of these findings emphasize the critical importance of weight management and body composition control in preventing premature aging. Particularly noteworthy is the potential intergenerational impact of maternal pre-pregnancy BMI on offspring LTL, highlighting the necessity for early intervention strategies in obesity prevention.

In this study, we combined 2SLS methodology with MR analysis to conduct subgroup analyses stratified by BMI categories and regional body fat distribution. Among the four primary obesity indices, BMI, BFP, and WC showed statistically significant associations with LTL, with each explaining 11%, 14%, and 12% of the variance in LTL, respectively. Notably, through subgroup analysis, we found that the inverse causal relationship between BMI and LTL was most pronounced in class Ⅲ obesity (BMI > 40 kg/m²), accounting for over 50% of LTL variance. This suggests that severe obesity may directly impact cellular aging through accelerated telomere shortening. For individuals with BMI < 40 kg/m², MR analysis revealed significance only in obesity class Ⅰ, although 2SLS analysis showed no significant differences across overweight, obesity class Ⅰ, and obesity class Ⅱ subgroups. Importantly, obesity class Ⅰ and Ⅱ each explained approximately 10% of LTL variation, suggesting that the impact of obesity (BMI > 30 kg/m²) on cellular aging remains noteworthy despite varying statistical significance. Interestingly, overweight status (BMI 25 ~ 29.9 kg/m²) demonstrated neither causal relationship with LTL shortening nor substantial explanatory power (< 3% of LTL variation). These findings emphasize the critical importance of targeted weight management and early intervention strategies, particularly for individuals with severe obesity, to mitigate accelerated telomere shortening and its associated health consequences.

Our study reveals a causal relationship between BFP and LTL, with consistent negative effects observed across all regional fat depots. Notably, both limb fat and trunk fat demonstrated significant causal associations with telomere shortening, though with different explanatory power: limb adiposity explained over 15% of LTL variation while trunk fat accounted for 8%. These findings indicate that regardless of anatomical location, fat accumulation may have the potential to accelerate cellular aging. The pervasive negative impact of regional fat deposits on telomere length suggests a shared underlying mechanism. Adipose tissue, regardless of its distribution, serves as an active endocrine organ that secretes pro-inflammatory cytokines and adipokines^72^. As fat accumulates in different body regions, it creates a state of chronic low-grade systemic inflammation^73,74^. This chronic inflammatory milieu may accelerate telomere attrition through multiple pathways: increased oxidative stress that damages telomeric DNA, impaired telomerase activity due to inflammatory signaling, and enhanced cellular senescence in response to persistent inflammatory stimuli^75,76^. Therefore, chronic inflammation may emerge as a potential key mechanism linking regional fat distribution to telomere shortening. This inflammatory hypothesis explains why fat accumulation in any anatomical location—whether metabolically “favorable” subcutaneous fat or “unfavorable” visceral fat—may ultimately contribute to cellular aging. These insights emphasize that effective interventions for preventing cellular aging should target overall fat reduction and inflammation control, rather than focusing solely on specific fat depot redistribution.

Through MVMR analysis, we examined the independence of obesity’s effects by adjusting for major metabolic-related phenotypes (lipid profiles and T2D). Notably, the causal associations of BMI and BFP with LTL remained statistically significant after these adjustments, while the effects of WC and WHR were attenuated. These findings establish BMI and BFP as independent risk factors for telomere shortening, operating through pathways distinct from traditional metabolic factors. BMI and BFP, as indicators of general obesity, appear to influence telomere dynamics through pathways that are largely independent of lipid metabolism and glucose homeostasis. This suggests that general adiposity may affect cellular aging through mechanisms such as systemic inflammation, oxidative stress, or hormonal dysregulation that operate beyond traditional cardiometabolic pathways^77^. In contrast, WC effects were attenuated after metabolic adjustment, indicating that waist circumference’s impact on LTL is largely mediated through metabolic pathways. WHR showed no significant association in either univariate or multivariate analyses, suggesting that relative fat distribution patterns may not directly influence telomere dynamics. This finding aligns with the established role of visceral fat in driving insulin resistance, dyslipidemia, and inflammatory processes. Interestingly, in our univariate analyses, WC showed a significant negative causal association with LTL while WHR did not, despite both being established predictors of cardiometabolic risk. This difference likely reflects what these measures capture biologically—WC directly reflects absolute visceral fat mass, whereas WHR represents relative fat distribution patterns that may be influenced by individual variations in body structure and muscle mass. These mechanistic distinctions have important clinical implications. Our findings suggest that interventions targeting general obesity (BMI/BFP reduction) may provide cellular aging benefits through metabolic-independent pathways, while visceral fat reduction strategies may primarily benefit telomere health through improvements in metabolic function.

Our transcriptomic analysis revealed significant effects of obesity on immune cell senescence, supported by multiple lines of evidence. First, we identified aberrant expression of established senescence markers, including *ID2*^78^, *CDKN1A*^79,80^, *TENT4B*, and *LMNA*^81–83^. Moreover, we observed elevated expression of IL6, a key component of the senescence-associated secretory phenotype (SASP)^84–86^, in individuals with obesity, further supporting obesity’s role in accelerating immune cell senescence. Gene enrichment analysis revealed significant alterations in immune cell functions, particularly in pathways related to cell adhesion, activation, and molecular binding. Inflammatory response pathways were markedly upregulated in individuals with obesity, underscoring the key role of obesity in aging-related immune dysregulation. Following bariatric surgery, we observed significant downregulation of senescence-associated genes (*ID2*, *LMNA*, and *TENT4B*) and histone-related genes (*H2AC6*, *H2BC4*, *H2BC5*, *H2BC6*, and *H2AC20*), suggesting that epigenetic modulation may influence the aging process through surgical intervention. Interestingly, *ID2* was upregulated in PBMCs from obese individuals but downregulated after bariatric surgery. This contrasts with previous findings reporting *ID2* downregulation as a hallmark of aging in non-immune cells via *NRF2*-mediated antioxidant pathways^77^. Such divergence may reflect cell-type-specific regulation of *ID2* under chronic metabolic stress. In immune cells, *ID2* upregulation may represent a protective or compensatory response to obesity-induced inflammation. However, the precise functional role of *ID2* in immunosenescence and its connection to telomere dynamics warrants further investigation. Furthermore, the observed reduction in histone gene expression after surgical intervention suggests that changes in chromatin structure and histone modification may represent a key mechanism through which obesity-related aging processes can be reversed. This is consistent with prior studies identifying aberrant histone modifications as a hallmark of aging^87,88^. Our study revealed that bariatric surgery may reverse obesity-induced accelerated aging effects through the remodeling of histone expression profiles. These epigenetic alterations not only help explain the overall physiological improvements observed in post-surgical patients but also provide new insights into developing interventional strategies targeting obesity-related aging. Moreover, we also observed a significant downregulation of *CH25H* in PBMCs following bariatric surgery. As an interferon-inducible enzyme, *CH25H* participates in inflammatory responses^89,90^ and metabolic regulation through catalyzing the conversion of cholesterol to 25-hydroxycholesterol^91,92^. The decreased expression not only reflects the improvement of systemic inflammatory status post-surgery but also indicates a metabolic reprogramming of lipid metabolism. Furthermore, given the crucial role of *CH25H* in immune cell function regulation, this finding provides new molecular mechanistic insights into the improvement of the immune microenvironment following bariatric surgery. Although inflammatory-related gene sets remained partially activated, these findings demonstrate that obesity-induced immune cell senescence can be potentially reversed through weight management interventions. This reversibility underscores the critical importance of addressing obesity in the context of immune system aging and function.

The strength of this study is derived from the utilization of univariate and multivariate MR analysis, which bolster the reliability of the findings and mitigate the potential confounding effects of metabolic status. Furthermore, sensitivity analysis revealed no substantial heterogeneity or pleiotropy in the IVs employed, thereby affirming the validity of the conclusions. The incorporation of the 2SLS method in this study facilitated a more precise estimation of the causal impact of exposure on outcomes, offering robust statistical evidence for elucidating the complex relationship between weight indices and LTL. The application of the 2SLS method enhances the statistical robustness of the results, while also helping to clarify the potential biological relevance of the observed associations^93^. However, this study has several important limitations. First, and most significantly, our analysis lacks a lean reference group (BMI < 25), with comparisons confined to overweight and obese individuals. This means our conclusions about obesity’s effects on LTL are relative to higher BMI categories rather than optimal health profiles. Without lean controls, we cannot establish whether observed effects represent pathological changes specific to excess adiposity or identify critical BMI thresholds where cellular aging accelerates, limiting the clinical interpretation of our findings. Second, due to data source constraints, we may not have captured all relevant factors that could influence the obesity-telomere relationship, including comprehensive metabolic hormones and inflammatory markers, which may be important mediators in this pathway. Third, our analysis included anatomically specific measurements (left/right limb fat) that may have limited clinical relevance, while lacking assessment of muscle mass or fat-free mass. This represents a significant limitation, as muscle quantity and quality are essential for understanding sarcopenia and metabolic health, and muscle loss is often a better predictor of adverse aging outcomes than fat mass alone. Fourth, while MR analysis provides robust causal inference, it relies on key assumptions that, if violated, could affect our conclusions. Although we used sensitivity analyses, potential confounding cannot be completely eliminated. Fifth, our findings may not be generalizable globally, as most GWAS data were derived from European ancestry populations, and we were unable to perform sex-stratified analyses due to pre-adjusted summary statistics. This limits our ability to explore sex differences, life-course influences, or population-specific effects systematically. Sixth, our analysis focused on LTL, which may not reflect telomere dynamics in other tissues. Future studies should incorporate comprehensive metabolic and muscle mass assessments, validate findings across diverse populations with lean control groups, and examine sex-specific effects when individual-level data are available.

## Conclusion

Through integrated MR and transcriptomic analyses, we established robust negative causal associations between obesity indices and LTL, with BMI and BFP effects persisting independently of metabolic factors. Transcriptomic analyses further revealed obesity-induced immune cell senescence through regulation of key genes (*ID2*, *LMNA*, *CDKN1A*), which could be reversed by bariatric surgery. This study reveals the molecular mechanisms linking obesity to accelerated biological aging and immunosenescence, providing compelling evidence for early clinical intervention in obesity management.

## Supplementary Information

Below is the link to the electronic supplementary material.

Supplementary Material 1

## Data Availability

Sequence data were deposited into the Gene Expression Omnibus database under accession number GSE32575 and are available at the following URL: https://www.ncbi.nlm.nih.gov/geo/query/acc.cgi? acc=GSE32575. GWAS data were deposited into the ieu OpenGWAS project (https://gwas.mrcieu.ac.uk/) and FinnGen database (https://risteys.finngen.fi/) under accession number listed in Figure 1.
