# Supplementary material for: Obesity impact on leukocyte telomere shortening and immune aging assessed by Mendelian randomization and transcriptomics analysis
Source: Sci Rep. 2025 Aug 23;15:30983. doi: 10.1038/s41598-025-16817-5 (PMC12373864; doi:10.1038/s41598-025-16817-5)
Supplement: Supplementary file 1 — Supplementary Material 1 [file 41598_2025_16817_MOESM1_ESM.zip › Supplementary materials/Figures/Figure S15 Funnel plot for secondary exposures.pdf]

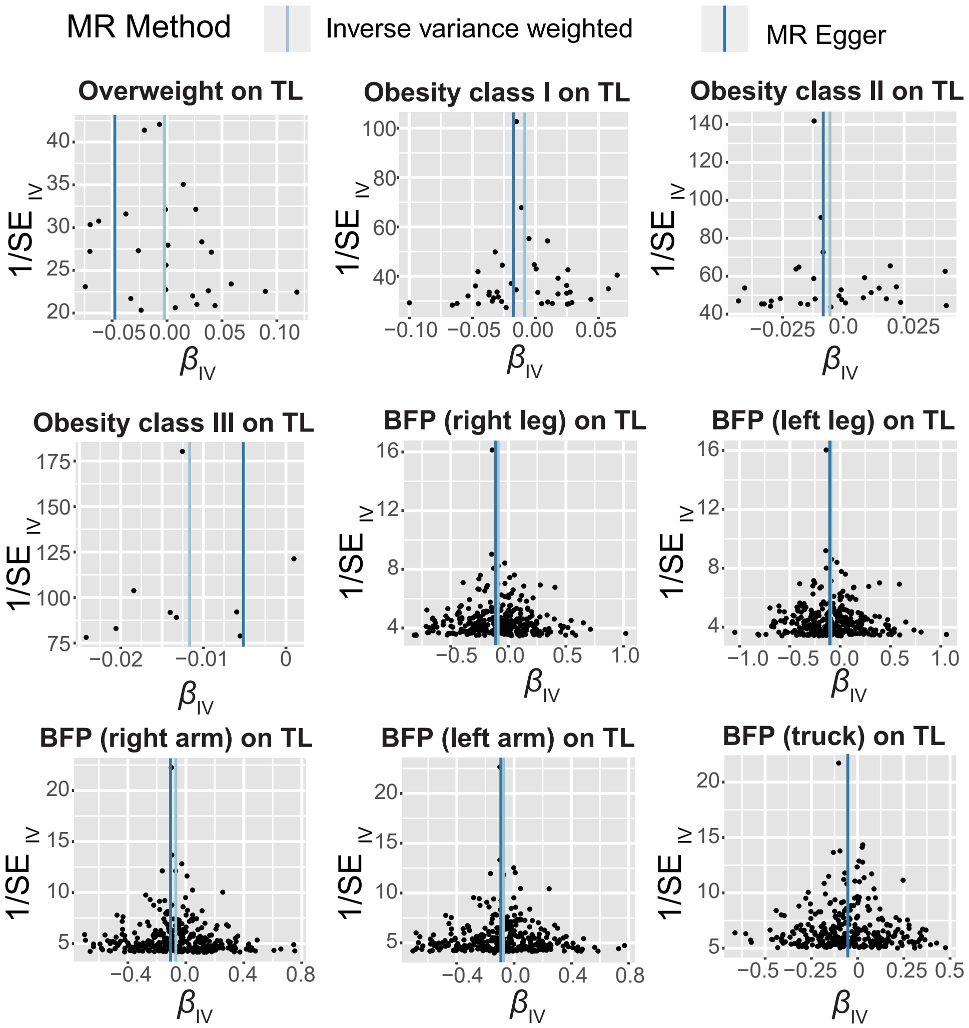
MR Method
Inverse variance weighted
MR Egger
Overweight on TL
Obesity class I on TL
Obesity class II on TL
Obesity class III on TL
BFP (right leg) on TL
BFP (left leg) on TL
BFP (right arm) on TL
BFP (left arm) on TL
BFP (truck) on TL
1/SE IV
β IV
