# Supplementary material for: Obesity impact on leukocyte telomere shortening and immune aging assessed by Mendelian randomization and transcriptomics analysis
Source: Sci Rep. 2025 Aug 23;15:30983. doi: 10.1038/s41598-025-16817-5 (PMC12373864; doi:10.1038/s41598-025-16817-5)
Supplement: Supplementary file 1 — Supplementary Material 1 [file 41598_2025_16817_MOESM1_ESM.zip › Supplementary materials/Figures/Figure S17 Leaveoneout_plot_of_MR_analysis_for ebi-a-GCST003435 to ieu-b-4879.pdf]

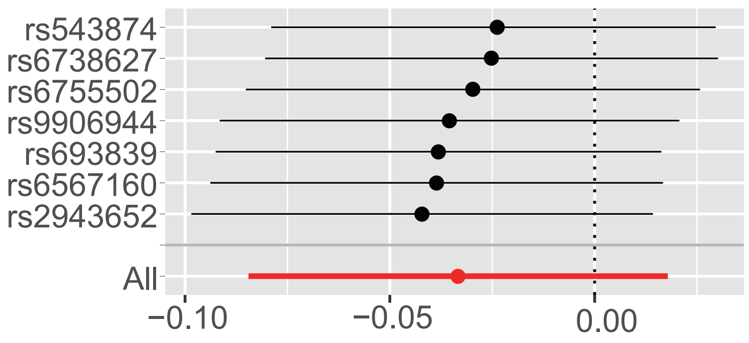

MR leave−one−out sensitivity analysis for
'Body fat percentage || id:ebi−a−GCST003435' on 'telomere length || id:ieu−b−4879'
