# Supplementary figures and images for: Obesity impact on leukocyte telomere shortening and immune aging assessed by Mendelian randomization and transcriptomics analysis
Source: Sci Rep. 2025 Aug 23;15:30983. doi: 10.1038/s41598-025-16817-5 (PMC12373864; doi:10.1038/s41598-025-16817-5)

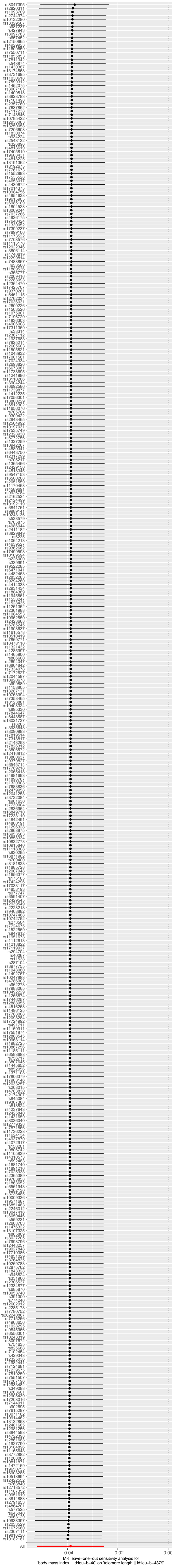

Supplement: Supplementary file 1 — Supplementary Material 1 [file 41598_2025_16817_MOESM1_ESM.zip › Supplementary materials/Figures/Figure S1 Leaveoneout_plot_of_MR_analysis_for_ieu-b-40_to_ieu-b-4879.pdf]

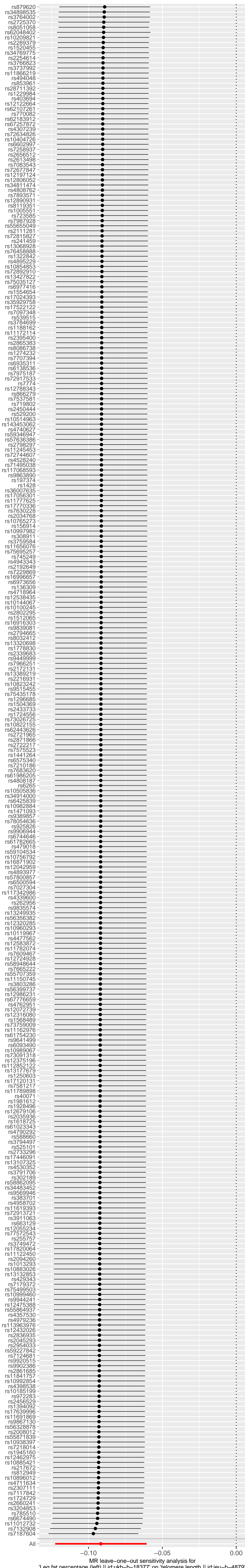

Supplement: Supplementary file 1 — Supplementary Material 1 [file 41598_2025_16817_MOESM1_ESM.zip › Supplementary materials/Figures/Figure S10 Leaveoneout_plot_of_MR_analysis_for_ukb-b-18377_to_ieu-b-4879.pdf]

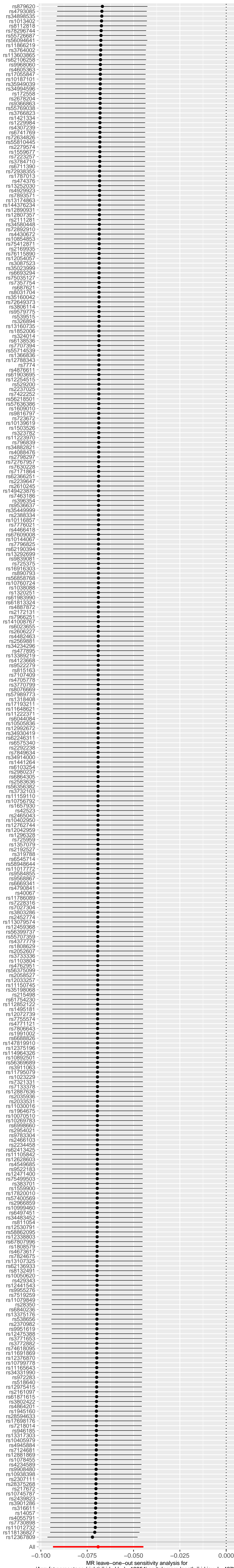

Supplement: Supplementary file 1 — Supplementary Material 1 [file 41598_2025_16817_MOESM1_ESM.zip › Supplementary materials/Figures/Figure S11 Leaveoneout_plot_of_MR_analysis_for_ukb-b-12854_to_ieu-b-4879.pdf]

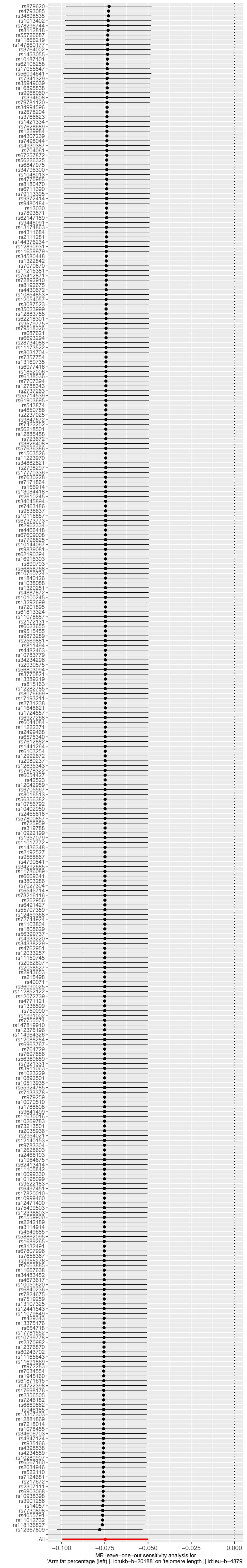

Supplement: Supplementary file 1 — Supplementary Material 1 [file 41598_2025_16817_MOESM1_ESM.zip › Supplementary materials/Figures/Figure S12 Leaveoneout_plot_of_MR_analysis_for_ukb-b-20188_to_ieu-b-4879.pdf]

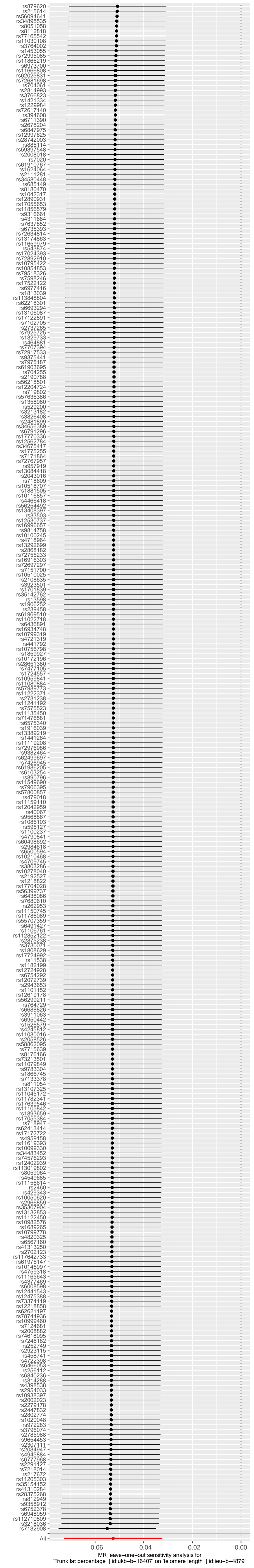

Supplement: Supplementary file 1 — Supplementary Material 1 [file 41598_2025_16817_MOESM1_ESM.zip › Supplementary materials/Figures/Figure S13 Leaveoneout_plot_of_MR_analysis_for_ukb-b-16407_to_ieu-b-4879.pdf]

MR Test   / IVW   / MR Egger   / Simple mode   / Weighted median   / Weighted mode

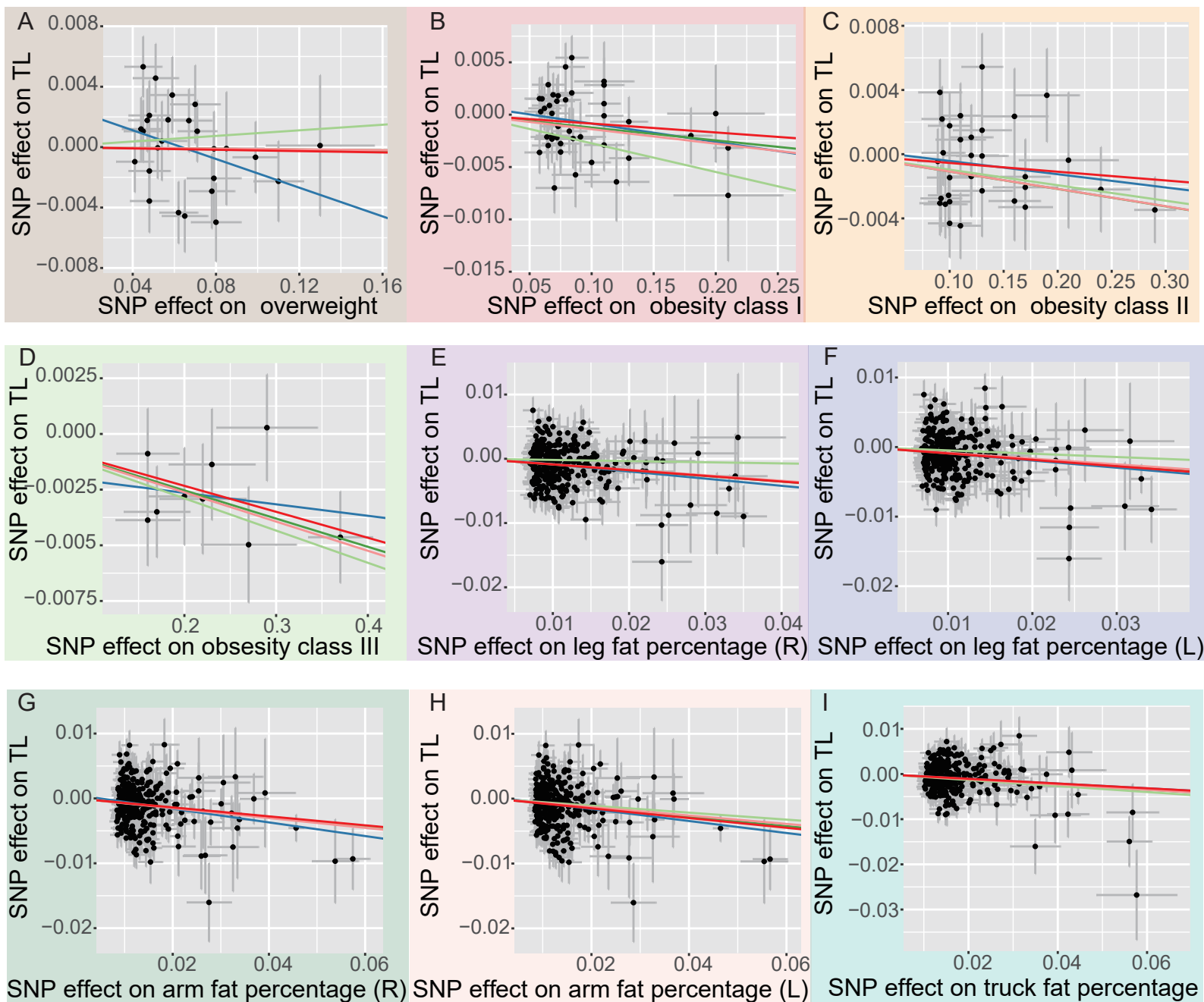

Supplement: Supplementary file 1 — Supplementary Material 1 [file 41598_2025_16817_MOESM1_ESM.zip › Supplementary materials/Figures/Figure S14 Scatter plot for secondary exposures.pdf]

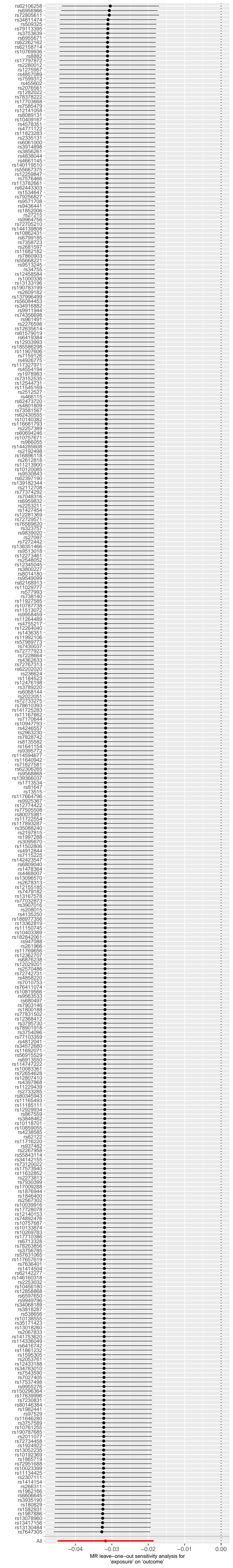

Supplement: Supplementary file 1 — Supplementary Material 1 [file 41598_2025_16817_MOESM1_ESM.zip › Supplementary materials/Figures/Figure S16 Leaveoneout_plot_of_MR_analysis_for_finngen_R10_BMI_IRN_to_ieu-b-4879.pdf]

CC

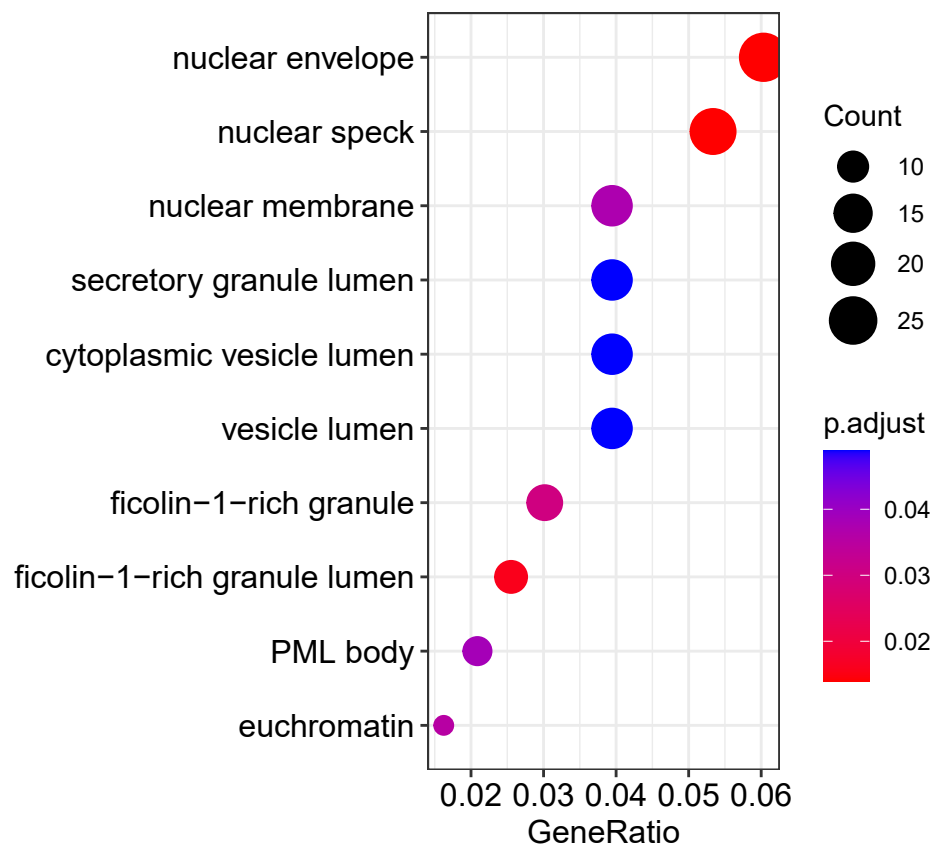

BP

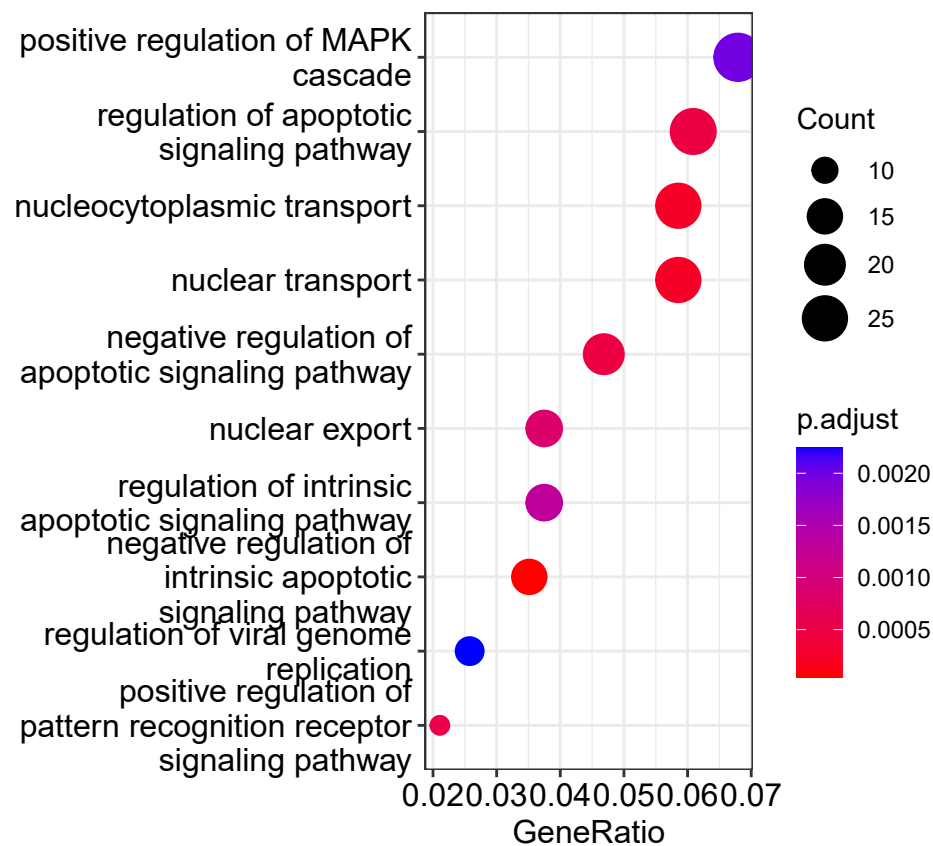

MF

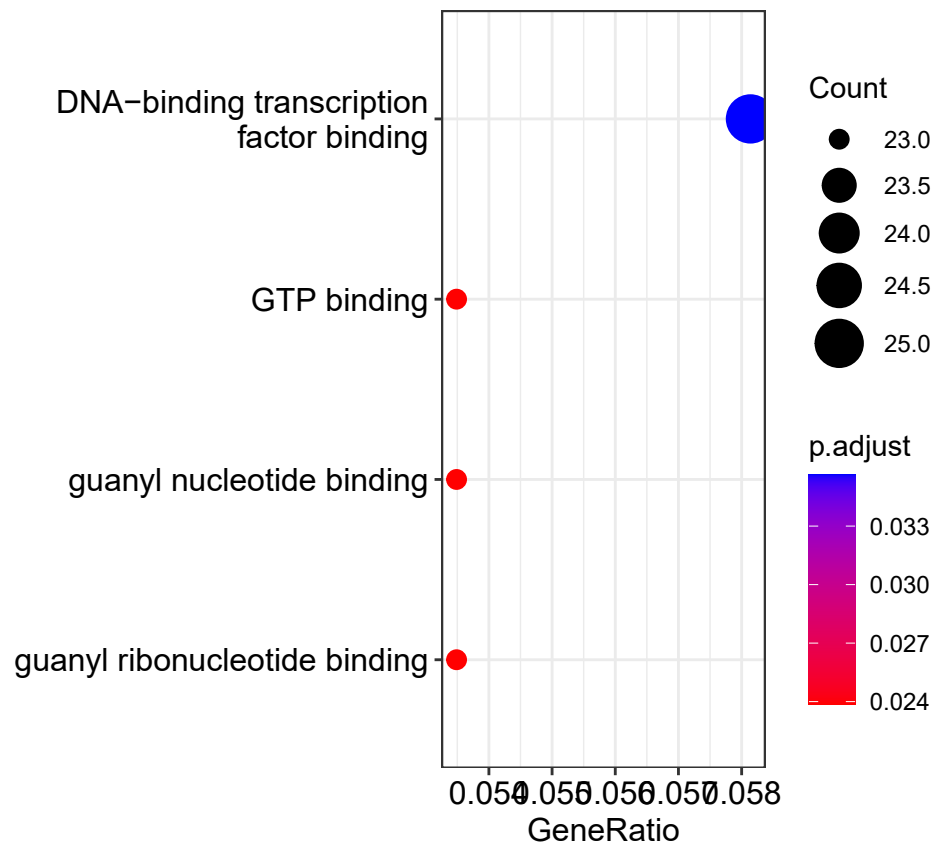

KEGG

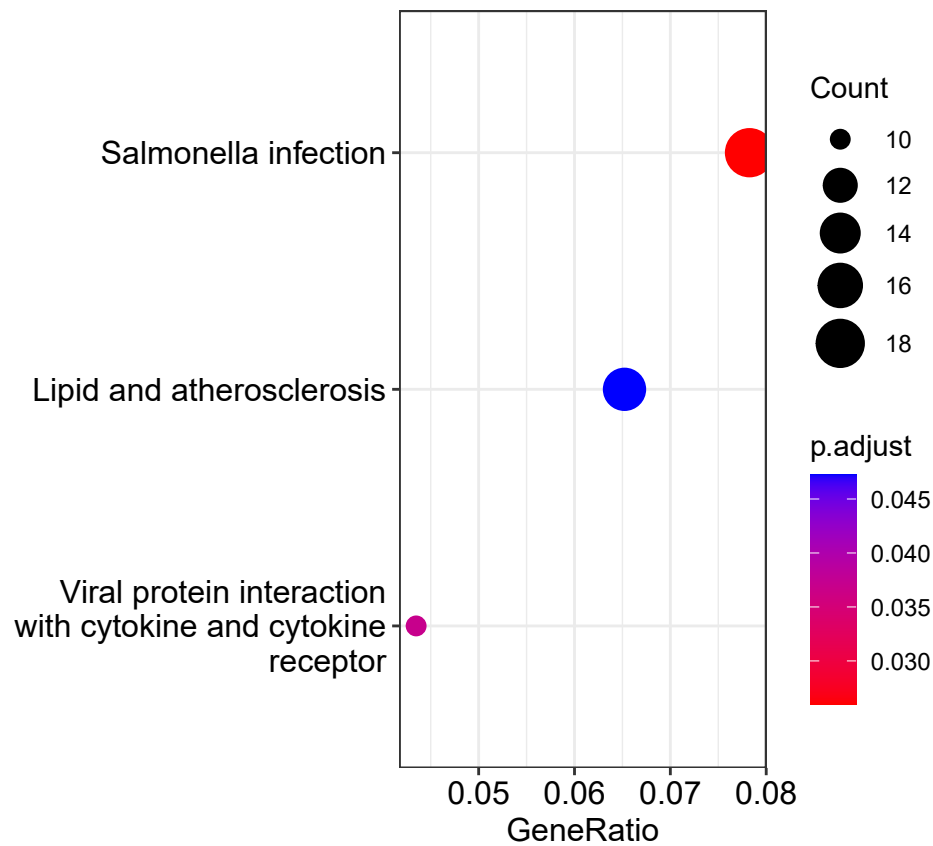

Supplement: Supplementary file 1 — Supplementary Material 1 [file 41598_2025_16817_MOESM1_ESM.zip › Supplementary materials/Figures/Figure s18 GO and KEGG enrichment analysis for DEGs between before surgery and after surgery.pdf]

KEGG\_GSEA

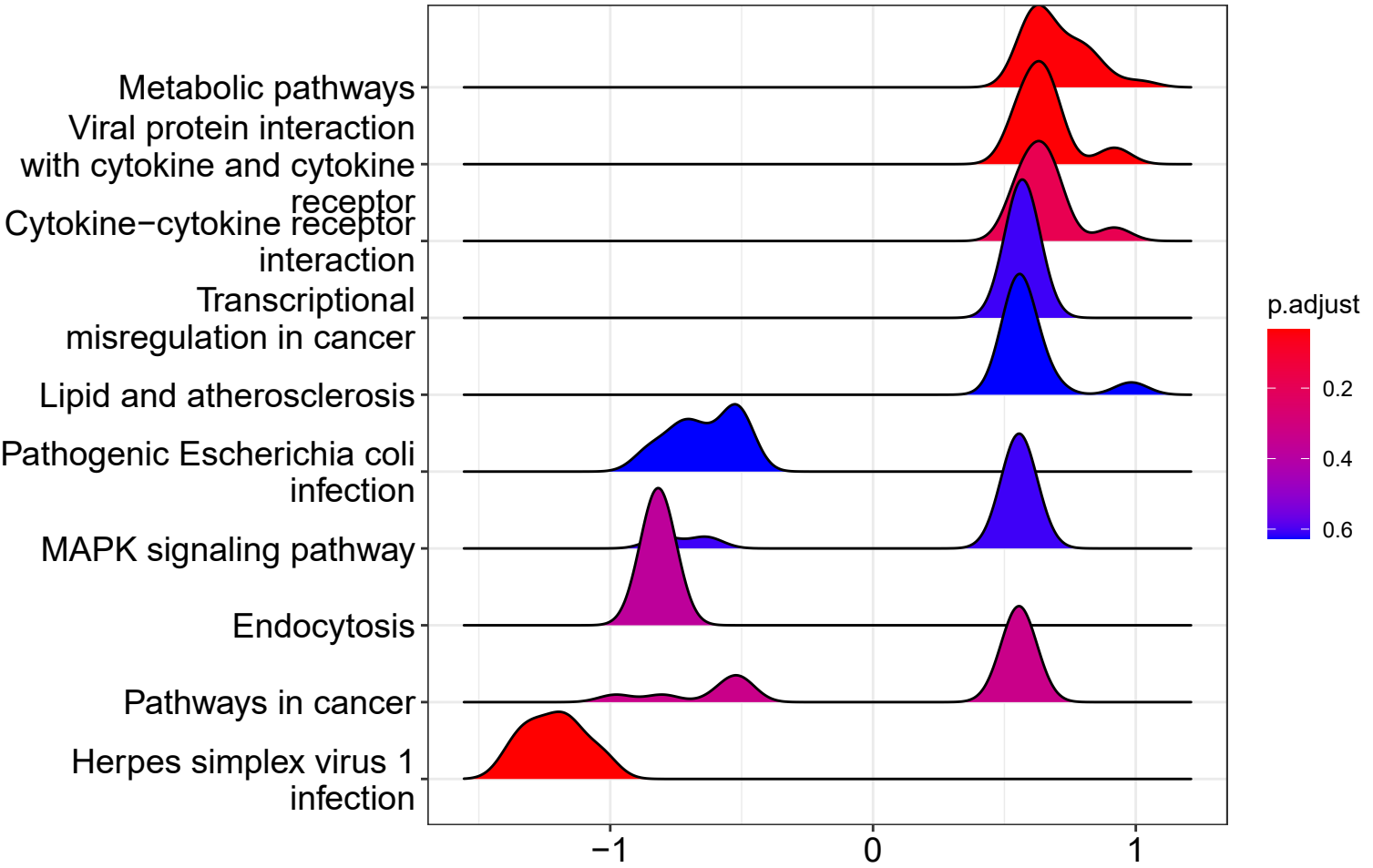

Reactome\_GSEA

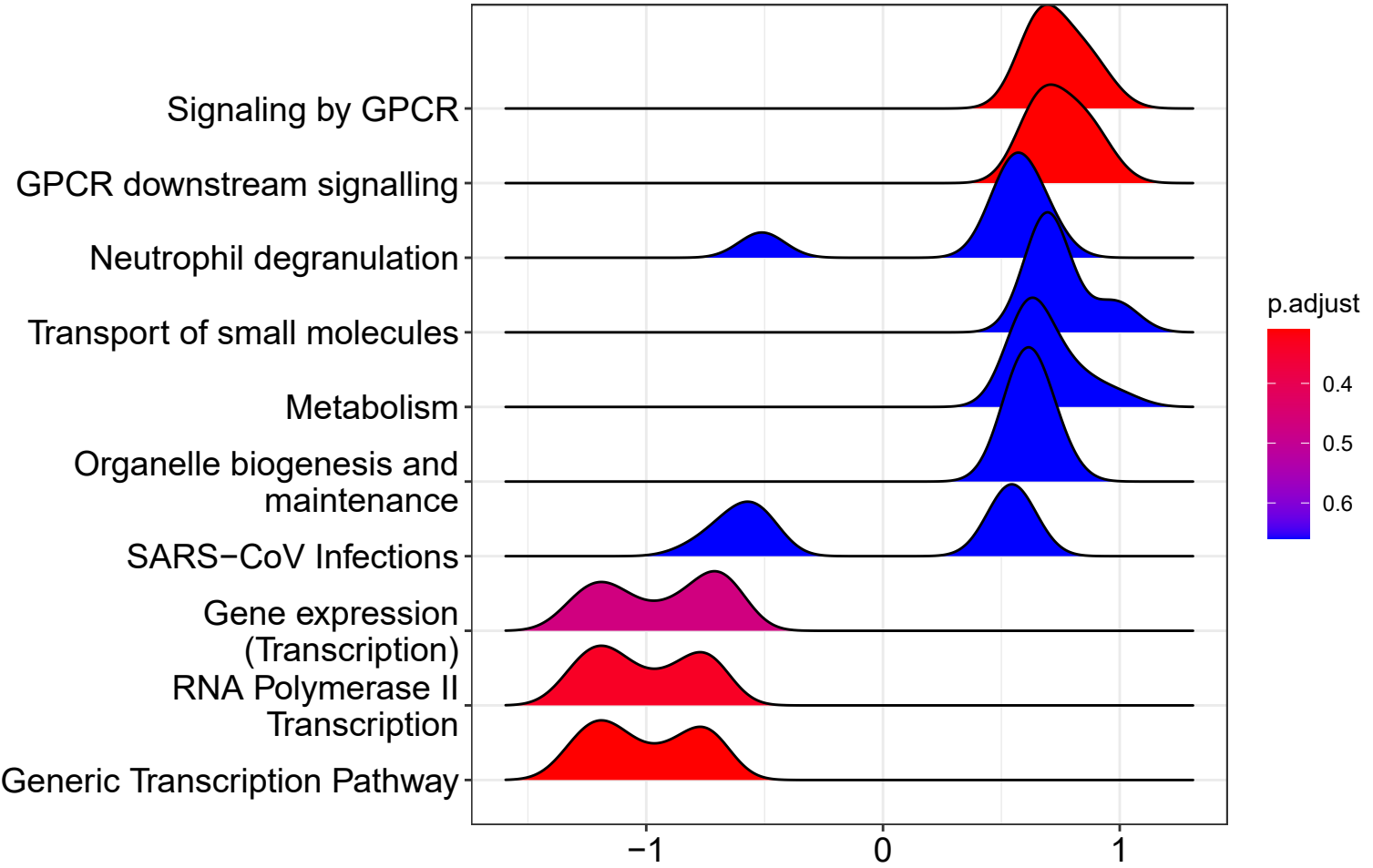

Supplement: Supplementary file 1 — Supplementary Material 1 [file 41598_2025_16817_MOESM1_ESM.zip › Supplementary materials/Figures/Figure s19 GSEA enrichment analysis for DEGs between before surgery and after surgery.pdf]

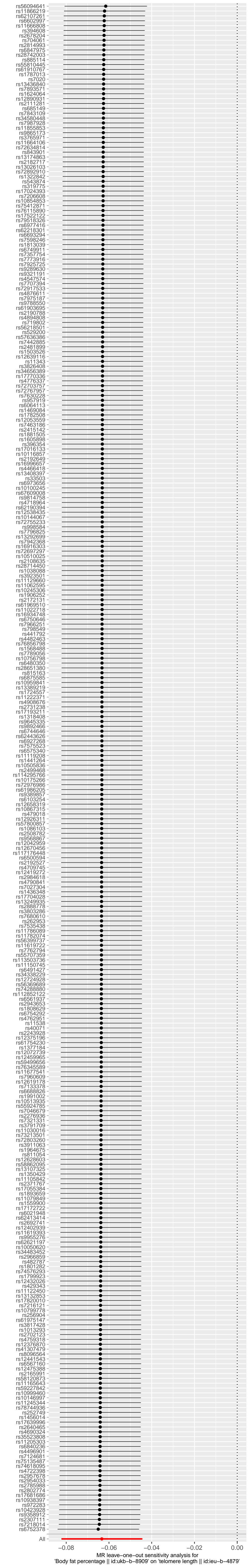

Supplement: Supplementary file 1 — Supplementary Material 1 [file 41598_2025_16817_MOESM1_ESM.zip › Supplementary materials/Figures/Figure S2 Leaveoneout_plot_of_MR_analysis_for_ukb-b-8909_to_ieu-b-4879.pdf]

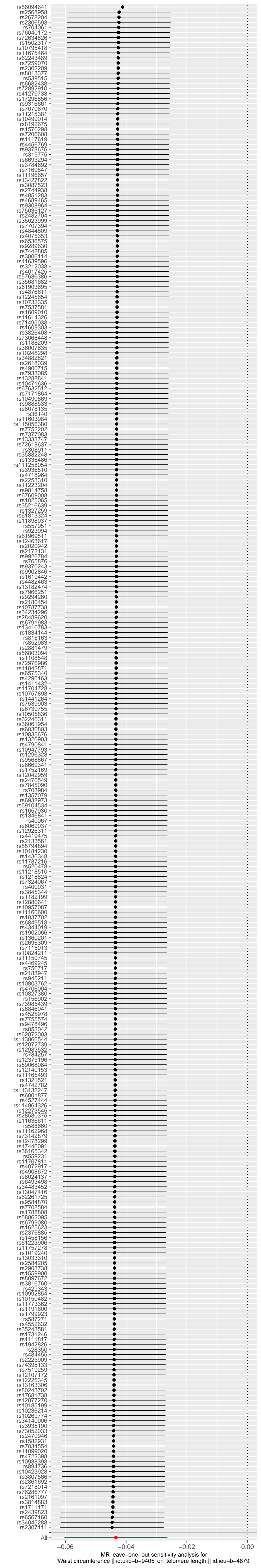

Supplement: Supplementary file 1 — Supplementary Material 1 [file 41598_2025_16817_MOESM1_ESM.zip › Supplementary materials/Figures/Figure S3 Leaveoneout_plot_of_MR_analysis_for_ukb-b-9405_to_ieu-b-4879.pdf]

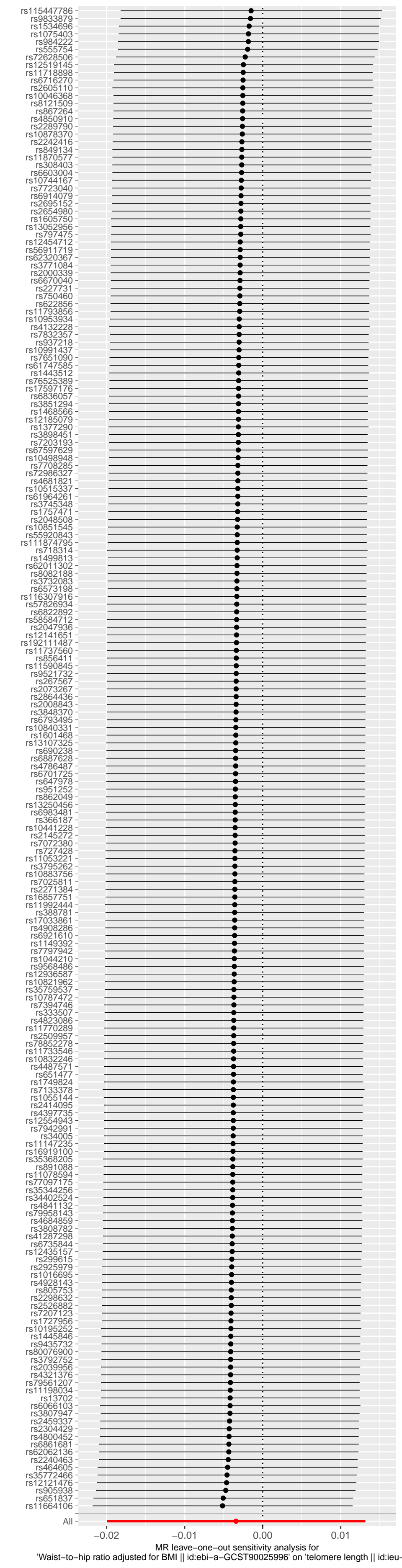

Supplement: Supplementary file 1 — Supplementary Material 1 [file 41598_2025_16817_MOESM1_ESM.zip › Supplementary materials/Figures/Figure S4 Leaveoneout_plot_of_MR_analysis_for_ebi-a-GCST90025996_to_ieu-b-4879.pdf]

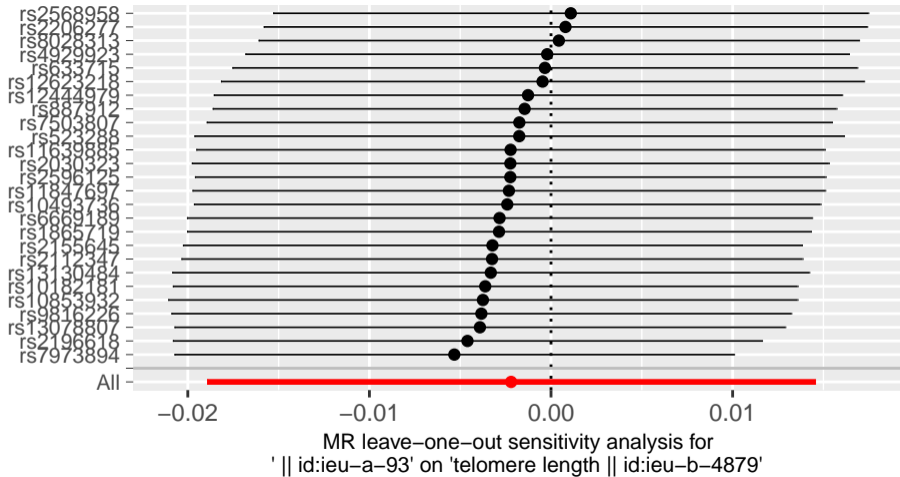

Supplement: Supplementary file 1 — Supplementary Material 1 [file 41598_2025_16817_MOESM1_ESM.zip › Supplementary materials/Figures/Figure S5 Leaveoneout_plot_of_MR_analysis_for_ieu-a-93_to_ieu-b-4879.pdf]

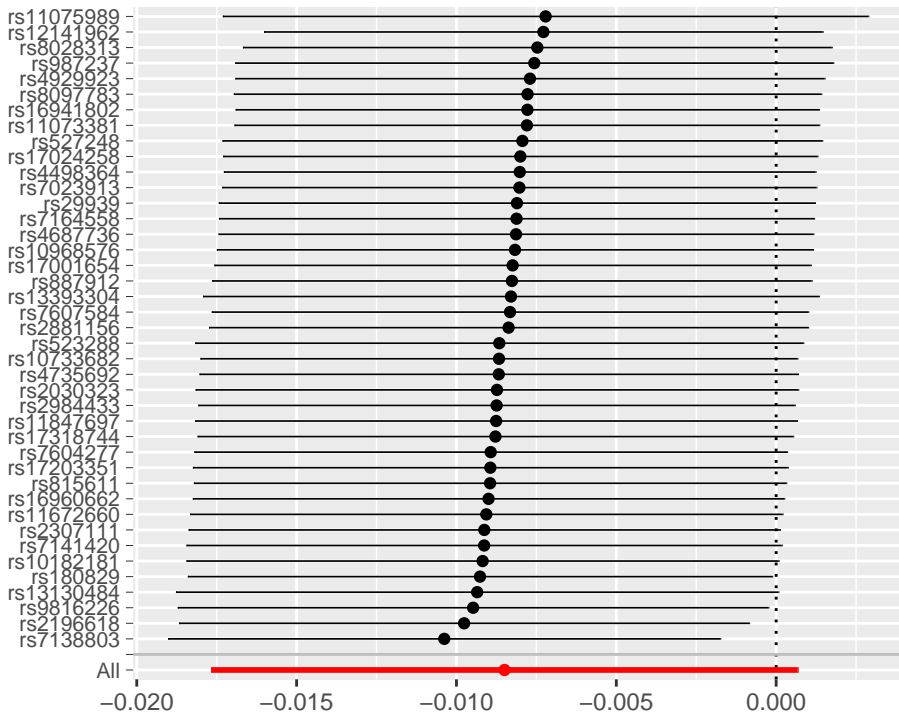

MR leave-one-out sensitivity analysis for  
' || id:ieu-a-90' on 'telomere length || id:ieu-b-4879'

Supplement: Supplementary file 1 — Supplementary Material 1 [file 41598_2025_16817_MOESM1_ESM.zip › Supplementary materials/Figures/Figure S6 Leaveoneout_plot_of_MR_analysis_for_ieu-a-90_to_ieu-b-4879.pdf]

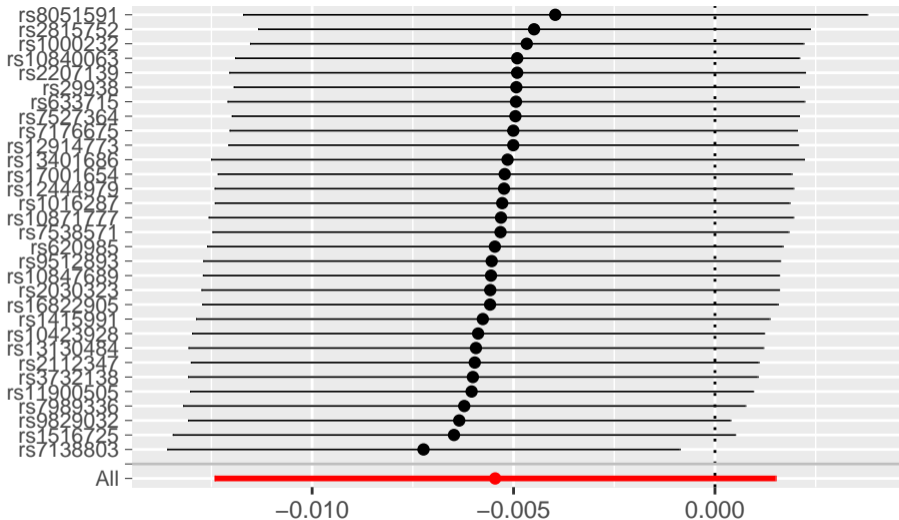

Supplement: Supplementary file 1 — Supplementary Material 1 [file 41598_2025_16817_MOESM1_ESM.zip › Supplementary materials/Figures/Figure S7 Leaveoneout_plot_of_MR_analysis_for_ieu-a-91_to_ieu-b-4879.pdf]

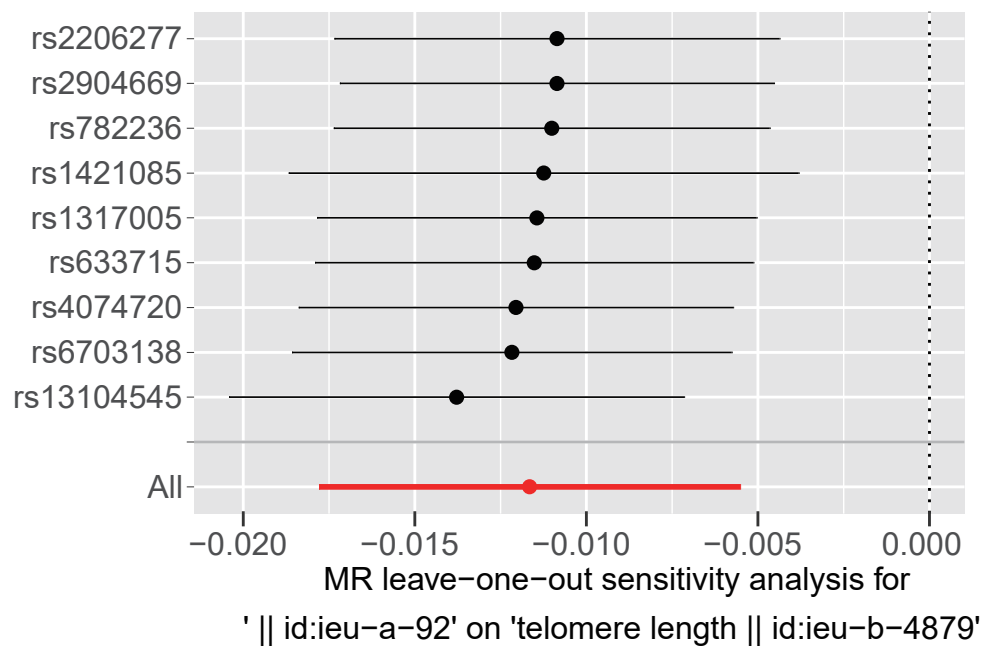

Supplement: Supplementary file 1 — Supplementary Material 1 [file 41598_2025_16817_MOESM1_ESM.zip › Supplementary materials/Figures/Figure S8 Leaveoneout_plot_of_MR_analysis for ieu-a-92 to ieu-b-4879.pdf]

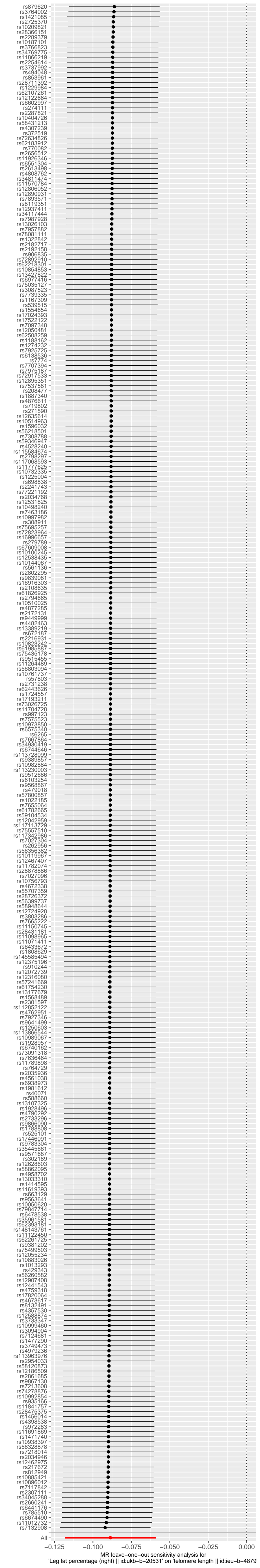

Supplement: Supplementary file 1 — Supplementary Material 1 [file 41598_2025_16817_MOESM1_ESM.zip › Supplementary materials/Figures/Figure S9 Leaveoneout_plot_of_MR_analysis_for_ukb-b-20531_to_ieu-b-4879.pdf]
